# Feasibility and Methodological Reflections From the Co-Creation of a Randomized Controlled Trial (The Kid’s Trial): Decentralized, Child-Led Citizen-Science Study

**DOI:** 10.2196/89240

**Published:** 2026-08-28

**Authors:** Simone Lepage, Laura Flight, Nikki Totton, Declan Devane

**Affiliations:** 1 School of Nursing, Midwifery & Evidence Science, University of Galway, Áras Moyola, Upper Newcastle Rd, Galway, Ireland, +353 (0)91 493432; 2 HRB-Trials Methodology Research Network, University of Galway, Galway, Ireland; 3 National Institute for Health and Care Excellence, Manchester, England, United Kingdom; 4 Sheffield Centre for Health and Related Research (SCHARR), School of Medicine and Population Health, University of Sheffield, Sheffield, England, United Kingdom; 5 Evidence Synthesis Ireland, University of Galway, Galway, Ireland; 6 Cochrane Ireland, University of Galway, Galway, Ireland

**Keywords:** randomized controlled trial, children, health literacy, citizen science, decentralized trial, public involvement, patient and public involvement

## Abstract

**Background:**

Limited public understanding of randomized controlled trials (RCTs) hinders recruitment, retention, and confidence in research. Early exposure to trial concepts may strengthen health literacy and research engagement. The Kid’s Trial was a global, decentralized, child-led study that cocreated and conducted an RCT to help children understand trials and their importance and to improve critical thinking.

**Objective:**

This paper evaluated the feasibility and methodological implications of engaging children in the cocreation and conduct of a fully online RCT.

**Methods:**

The Kid’s Trial ed a dedicated website guiding children through each step of designing and conducting an RCT. Materials were codeveloped with 2 patient and public involvement groups of children and parents. Any child aged 7-12 years could take part in as many steps as desired. Recruitment combined online and offline strategies, and engagement and self-reported learning were descriptively analyzed. The cocreated Randomized Evaluation of Sleeping With a Toy or Comfort Item (REST) trial was a 2-arm, pragmatic RCT comparing one week of sleeping with a comfort item versus without a comfort item. The primary outcome was sleep-related impairment, and the secondary outcome was overall sleep quality. Analyses followed an intention-to-treat (ITT) approach using mixed effects models adjusted for baseline measures.

**Results:**

Overall, 224 children participated in at least one step of The Kid’s Trial. Participation varied: 37% (n=82) completed one step, and 21% (n=48) completed 6 surveys. The REST trial randomized 139 children, with 73% (n=101) completing outcome surveys. Adjusted mean differences (intervention – control) were −0.53 (95% CI −3.40 to 2.34) for sleep-related impairment (*P*=.71) and 0.28 (95% CI 0.01-0.55) for sleep quality (*P*=.04). The difference was small and was not supported by sensitivity analyses. Poststudy responses (n=20) suggested improved self-reported trial understanding among respondents but were limited by low response rate and potential selection bias.

**Conclusions:**

The Kid’s Trial demonstrates the feasibility of a decentralized, child-led RCT cocreated through participatory citizen-science methods. Children can meaningfully contribute to trial design and conduct, and experiential participation may support engagement with trial concepts. Future studies should enhance engagement through community partnerships, shorter intervals between steps, and embedded learning assessments to improve inclusivity and retention.

## Introduction

Randomized controlled trials (RCTs) are widely regarded as the gold standard for evaluating the effectiveness of health care interventions. By minimizing bias, they can generate reliable evidence to guide clinical and policy decisions [1]. Despite their importance, RCTs face persistent challenges, including difficulties in recruiting and retaining participants, complex logistics, and high operational costs [2,3]. A limited public understanding of trials contributes to these challenges [4,5].

Evidence suggests that when members of the public understand the purpose and processes of RCTs, they are more likely to support and participate in them [5,6]. This has prompted researchers to explore ways to educate and engage the public in research. For example, in 2010, a Scottish collaboration launched a media campaign on television, radio, and newspapers featuring clinical researchers, general practitioners, and patients discussing the importance of RCTs [7]. The organization “Clinical Trials for All,” established in 2023, is a noncommercial initiative aiming to “increase global clinical trial awareness and participation by educating patients and caregivers about the opportunities and advantages of clinical research as a care option” [8].

While these initiatives are valuable, approaches that involve the public directly in the research process may offer a more experiential way to build understanding of how RCTs are designed, conducted, and used. The People’s Trial, launched in 2019, involved over 3000 adults from 72 countries in the design and conduct of an online RCT led by the public [9]. By making the research process participatory and transparent, the initiative successfully heightened public awareness and interest in clinical trials [9]. However, there is limited evidence on how this participatory model can be applied with children, particularly in fully online settings.

The Kid’s Trial was developed to address this gap by extending the participatory model used by The People’s Trial to children aged 7-12 years. The Kid’s Trial was conducted entirely online, and children were invited to take part in all aspects of a randomized trial, from generating questions and designing the study to participating in the trial they designed and deciding how to share the results. The Kid’s Trial aimed to help children learn what RCTs are and why they matter by actively involving them in creating and running one, while encouraging them to think critically about the health information they encounter.

Focusing on children during middle childhood is relevant in this context because this developmental stage is marked by growing reasoning abilities, curiosity, and an openness to new ideas [10,11]. It is also a critical period for laying the foundations of health literacy, that is, the ability to access, understand, and appraise health information to make informed decisions [12,13]. In today’s digital information landscape, where children are frequently exposed to numerous health claims, early exposure to evidence-based thinking is especially valuable [14].

The Kid’s Trial adopted a decentralized design, with all its trial activities conducted remotely using digital platforms. Decentralization includes strategies such as data collection via mobile devices or remote participation [15], which can lessen participant burden, enhance access and inclusivity, and improve the efficiency of clinical research [16-18]. We incorporated active public and patient involvement (PPI) by creating 2 online advisory groups of children and parents to shape the study’s direction and increase its relevance.

**Figure 1.**
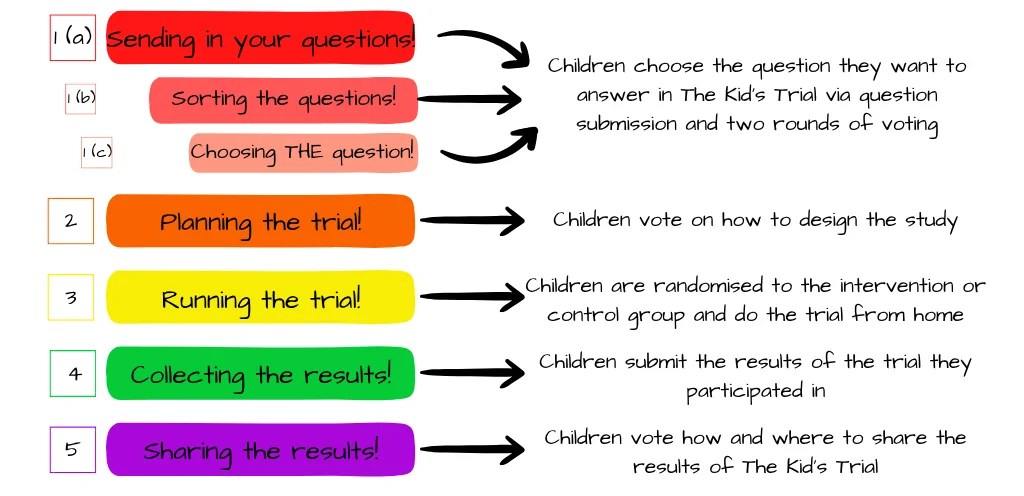
Steps of The Kid’s Trial.

To reflect the structure of a clinical trial, The Kid’s Trial was divided into a series of participatory steps that guided children through the research process, from choosing the question to sharing the results, as shown in Figure 1. Children contributed to each step of the process, including selecting the research question, defining trial procedures, taking part in the trial and reporting their results, and deciding how the findings would be shared.

This study reports on The Kid’s Trial as a global, decentralized, child-led citizen science study. We use the term citizen science to describe the participatory, child-led approach we used. The primary objective of The Kid’s Trial was to evaluate the feasibility and methodological implications of engaging children in the cocreation and conduct of a fully online RCT. The study included an embedded proof-of-concept RCT, the Randomized Evaluation of Sleeping With a Toy or Comfort Item (REST) trial, which is presented here to illustrate the implementation of this approach. In doing so, this work demonstrates how children can meaningfully engage with core trial processes and considers whether this participation may support children’s self-reported understanding of how trials are designed, conducted, and interpreted.

## Methods

### Ethical Considerations

The University of Galway Research Ethics Committee granted ethical approval for this study on January 16, 2023 (Ref: 2023.02.014).

#### Study Setting and Design

Children taking part in The Kid’s Trial could join from anywhere, with all trial materials and information available on the website [19]. To take part in any step of the study, children needed internet access via a computer, tablet, or mobile phone. Participation was intentionally flexible to align with the participatory citizen-science nature of the initiative, allowing children to join as many or as few steps as they wished. The Kid’s Trial ran for 19 months, from March 11, 2024, to October 9, 2025.

### PPI Groups

In this paper, we use the term PPI to refer specifically to the advisory groups that informed the study’s design and conduct. Eight months before launching The Kid’s Trial, we established a Children’s Research Advisory Group (CRAG) and a Parents’ Research Advisory Group (PRAG), comprising 7 family units (8 children aged 7-11 years and 7 parents). Child literacy levels and parent education levels were not collected for PPI group members. These groups were recruited using social media and email campaigns. Parent-child dyads were recruited to support participation in a fully online setting, particularly for younger children. Meetings were held online to accommodate the geographic diversity of participating families (Estonia, Ireland, Kenya, Norway, Tanzania, and the United States).

Over approximately 18 months, members of the CRAG and PRAG collaborated with the research team to refine the visual identity and tone of The Kid’s Trial website, codevelop age-appropriate consent and assent materials, shape the content and language of the animated explainer videos, and review survey wording for clarity and accessibility. Engagement was maintained through regular online meetings, structured feedback surveys, and anonymous polls. A detailed account of the activities, challenges, facilitators, and experiences of both PPI group members and researchers is provided in a separate report [20].

### Website and Survey Development

#### Overview

The website served as the central hub for the study. It provided study information, introduced the research team, explained the involvement of the PPI groups, outlined data and privacy policies, and presented each step. Each step’s webpage described its purpose, featured an animated explainer video, and linked participants to the corresponding survey. Surveys were accessed via a button that redirected to QuestionPro (QuestionPro Inc) survey software [21]. The steps were opened sequentially, with survey links closing once each step was completed. The website’s visual identity, including the logo and icons created by CRAG members, color scheme, fonts, and language, was co-designed with our PPI groups.

#### Animated Videos

To help maintain engagement and accessibility, we created animated explainer videos for children and their parents using Powtoon (Powtoon.com, Inc) software [22]. The research team drafted transcripts, developed animations, and shared drafts with the PPI groups for review. PPI feedback focused on both the animations and the language, and the groups requested an additional video explaining the concept of randomization. Children from the CRAG and our wider network narrated the videos. Ten videos were produced, introducing core concepts of RCTs and explaining the purpose of each step of The Kid’s Trial, what participation involved, and how to take part. These animated videos were uploaded to the website and the study’s YouTube (Google LLC) channel [23].

#### Recruitment

Recruitment was conducted using a combination of online and offline strategies, primarily aimed at parents. Online recruitment included social media posts on X (X Corp), Facebook (Meta Platforms, Inc), Instagram (Meta Platforms, Inc), TikTok (TikTok Ltd), Mastodon (Mastodon gGmbH), and LinkedIn (LinkedIn Corporation); email campaigns; listings on 2 websites that connect the public with research opportunities: the European Citizen Science (ECS) platform [24] and Children Helping Science (CHS) [25]; and promotion through newsletters and blogs within European and American research and trial communities. Offline recruitment included radio interviews; distributing flyers in schools, markets, and play centers; and collaborating with children’s advocacy groups in countries where we lacked established networks. Recruitment efforts also sought to reach groups that are often underrepresented in research participation, including children from underserved communities, home-schooling networks, and Traveller community links.

Between March 11, 2024, and September 15, 2025, over 400 social media posts (both static and animated) were shared, approximately 1500 flyers were distributed, 2 national radio interviews were aired in Ireland, and more than 300 personal and professional contacts were emailed. During the third step, “Running the trial!” which had a target sample size (n=292), we expanded recruitment efforts by testing paid Google ads and running a 3-month paid Facebook campaign.

Recruitment was passive, requiring children and their caregivers to initiate contact with the trial via email, social media, or the website. The participant information leaflets (“Parents’ Informational Flipbook” and “Children’s Information Flipbook”) were available on the website. Participants were informed, both in the flipbooks and during the consent and assent processes, that they could withdraw from the study at any time without consequences and that their data could be deleted prior to data analysis.

#### Participants

Eligible participants were children aged 7-12 years who met the inclusion criteria, as confirmed through guardian consent and participant assent using QuestionPro [21], as approved by our research ethics committee. The Kid’s Trial was open globally, with no geographic exclusions. However, due to budget constraints, trial materials were only available in English. Because of the study’s decentralized nature, children without internet access were inherently excluded. The inclusion and exclusion criteria were as follows:

##### Inclusion Criteria

Eligible children had to meet the following inclusion criteria:

1. Children aged 7-12 years
2. Sufficient proficiency in English to understand trial materials
3. Access to the trial’s online platform
4. Guardian consent for participation

##### Exclusion Criteria

Children were only excluded if they were unable to understand the study and provide assent.

### Participant Demographics

At each step, children and parents were directed to the relevant page on the website, viewed the corresponding animation, and then invited to join that step by clicking a link to the associated QuestionPro [21] survey. Demographic data collected in these surveys included a parent’s email address, the child’s age, gender, country of residence, and ethnicity. The parents’ email address and the child’s age were required fields, while gender, country of residence, and ethnicity were optional and were collected to assess the study’s demographic and geographical reach, and, specifically, whether the decentralized design facilitated participation across different countries, age groups, and genders. Demographic data were collected for each survey except for the final 2, “Collecting the results!” and “Sharing the results!” as only previously participating children were invited to join those steps.

### Steps of the Kid’s Trial

**Figure 2.**
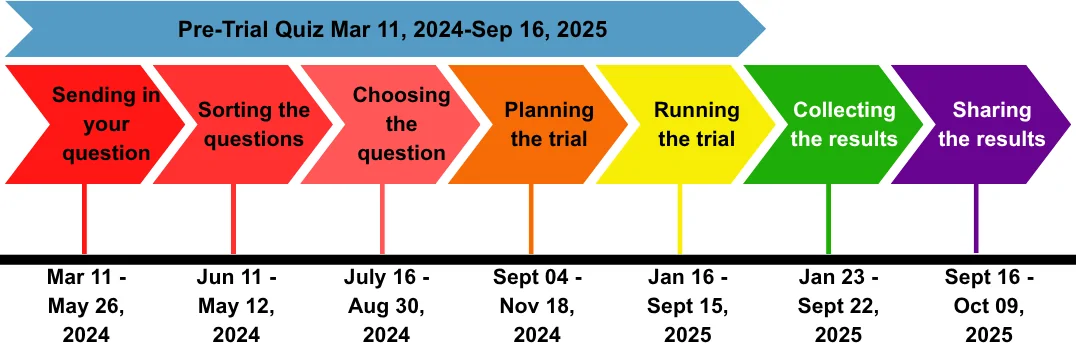
Timeline of The Kid’s Trial surveys.

This section summarizes the purpose and methods of each step of The Kid’s Trial*.* Figure 2 illustrates the sequence and timeline of these steps. The duration of each step varied depending on operational requirements: “Running the trial!” and “Collecting the results!” remained open for the longest period to facilitate recruitment for the embedded RCT, whereas “Sharing the results!” was necessarily concise due to project deadlines.

#### Pre-Trial Quiz: We Asked Children What They Already Knew About RCTs

The Pre-Trial Quiz included 3 simple questions about children’s prior knowledge of RCTs. Participation in this survey was not mandatory in the remaining steps of The Kid’s Trial, but children and parents were encouraged to complete this survey before proceeding to the other steps. This survey opened at the launch of The Kid’s Trial (March 11, 2024) and remained open until September 16, 2025. This survey is available in Multimedia Appendix 1.

#### Choosing the Question: a 3-Part Process

The first step of The Kid’s Trial aimed to identify which question we would try to answer with children worldwide. This step was divided into 3 parts. The 3 surveys that comprise the first step are available in Multimedia Appendix 2 (S2A sending in your questions survey, S2B sorting the questions survey, and S2C choosing the question survey).

##### Sending in Your Questions

**Figure 3.**
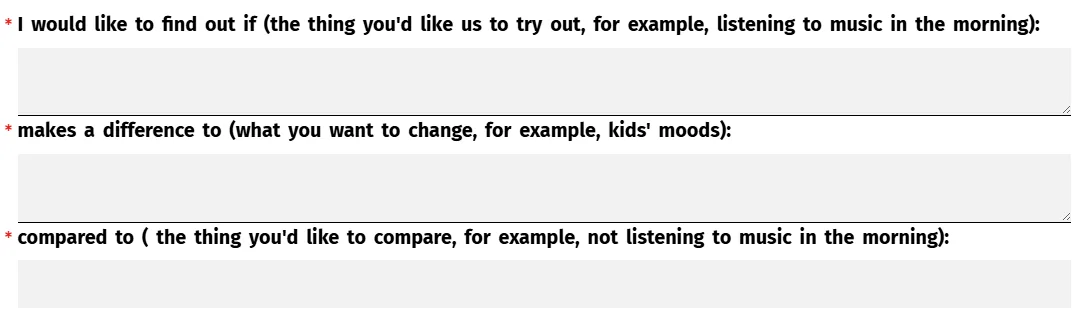
Survey 1 question submission form.

The first survey of The Kid’s Trial, “Sending in your questions!” was open from March 11, 2024, through May 26, 2024. This step invited children to submit fun, low-risk, health-related questions that they thought the study could address using a structured form (Figure 3). We provided examples of questions that could be tested, and children could use one of our examples or suggest their own.

After submissions closed, we carried out a 3-stage question prioritization process because not all questions could be considered for The Kid’s Trial. First, the research team reviewed each submitted question using the a priori inclusion criteria:

1. The question could not involve any medical interventions or testing, including dietary interventions.
2. The question was safe to test in an RCT, with no risks beyond children’s everyday activities.
3. The question did not require special equipment (eg, gaming devices and specialized sporting equipment).
4. The question would not financially burden a family.
5. The question could be tested and answered within a short timeframe (approximately 1 week).

Next, we shared our initial decisions with the PPI groups, asking for their agreement or reasons for disagreement regarding the inclusion/exclusion of specific questions. Finally, the research team reviewed any disagreements and the justifications provided, and reached consensus on which questions would advance to the next step.

##### Sorting the Questions

“Sorting the questions!” was open from June 11, 2024, through July 12, 2024. In this step, children told us how interested they were in answering the candidate questions that were submitted during the “Sending in your questions!” step. Their responses narrowed the list to 3 questions, which were carried forward into the final part of the first step: “Choosing the question!”

##### Choosing the Question

“Choosing the question!” opened on July 16, 2024, and closed August 30, 2024. In this step, children selected and ranked their top 3 questions to select the final trial question: *“*Does sleeping with a comfort item (for example, a soft toy or special blanket) make a difference to children’s sleep compared with not sleeping with a comfort item?” This question formed the basis of the randomized trial embedded within The Kid’s Trial, which was called the REST trial. The intervention group (ie, sleeping with a comfort item) was called the “Try-it-Out” group, while the control group (ie, sleeping without a comfort item) was the “Wait-and-See” group.

### Planning the Trial: Children Help Design the Trial

Between September 4, 2024, and November 18, 2024, children were invited to join the second step of The Kid’s Trial, in which children were asked to plan the trial that would address their selected question. They determined both the primary and secondary outcomes for the REST trial and the treatment arm procedures for participation in the “Try-it-Out” and “Wait-and-See” groups. To identify the outcomes, children were asked how sleep quality should be measured after the trial. They could select more than one of the following options:

1. Children completed an online survey the day after they finished their trial, reporting how their nighttime sleep was.
2. Children completed an online survey the day after they finished their trial, reporting how sleepy they felt during the day.
3. Children completed an online survey the day after they finished their trial, reporting how well they thought they had slept overall.

The 2 most popular options helped identify the primary and secondary outcomes for the REST trial. To identify the primary outcome, we asked which outcome best showed how children slept. Children selected daytime sleepiness as the primary outcome and overall sleep quality as the secondary outcome.

To define treatment and control arm procedures, the children were asked:

1. Should kids in both groups sleep in their usual beds for the entire trial?
2. Should kids in the “Try-it-Out” group use the same comfort item every night, or could they switch items?
3. When should kids in the “Try-it-Out” group start using their comfort item each night?

Children’s “usual beds” were defined as any bed where they regularly sleep, for example at home, at a relative’s house, or in another regular residence. The “Planning the trial!” survey is available in Multimedia Appendix 3

### Running the Trial: Children Join the Trial That They Created

In “Running the trial!” children were invited to take part in the REST trial. By clicking the “Join the trial here!” button on the website, children were redirected to a QuestionPro survey [21], in which baseline data on daytime sleepiness and overall sleep quality were collected before randomization. This step opened on January 16, 2025, and closed September 15, 2025.

Daytime sleepiness was measured using the Patient-Reported Outcomes Measurement Information System (PROMIS) Pediatric Short Form v1.0 Sleep-Related Impairment 4a [26], which yields a standardized T-score, with lower scores indicating less impairment [27,28]. Overall sleep quality was measured with the single-item Sleep Quality Scale (SQS) [29]. The SQS instrument consists of one question that asks participants to rate their overall sleep quality over the previous 7 days on a categorical scale coded 1‐5, with higher scores indicating better sleep quality [29].

The REST trial was a 2-arm, parallel-group, pragmatic superiority RCT. The intervention period lasted one week, and participants were randomized using a 1:1 ratio to either the “Try-it-Out” or “Wait-and-See” group using computer-generated randomization using QuestionPro [21]. Due to resource constraints, it was not possible to engage an independent researcher to clean and manage the data; consequently, the researchers became aware of group allocation after participant randomization. Because of the nature of the intervention, it was impossible to blind participants to their group. However, no party could influence or predict a participants’ group allocation. This survey is available in Multimedia Appendix 4.

### Collecting the Results: Children Tell Us the Results of Their Trial

In the fourth step, “Collecting the results!” parents of children who took part in the REST trial received an email with a survey link specific to their child’s allocated group 8 days after randomization. These surveys collected primary (sleep-related impairment [SRI]) and secondary (SQS) outcome data, along with additional group-specific questions on intervention adherence. These adherence data are reported in the REST trial report [30]. If the survey was not completed within 10 days postrandomization, a reminder email was sent, followed by a final reminder on day 13 postrandomization. These surveys are available in Multimedia Appendix 5 (S5A and S5B).

The REST trial protocol [31], statistical analysis plan (SAP) [32], and full trial report [30] detail the methods, analyses performed, and full results. Analyses followed an intention-to-treat (ITT) approach using a mixed effects model with treatment group as the main predictor, adjusting for baseline outcome, baseline comfort item use, age, and gender as fixed effects and country of residence as a random effect. Additionally, exploratory subgroup and sensitivity analyses were conducted as outlined in the SAP to assess differences by baseline characteristics.

### Sharing the Results: Children Decide How and Where to Share the Results of the Trial

In “Sharing the results!” all parents whose children had previously participated in any step of The Kid’s Trial received an email inviting their children to rank different methods and locations in which to share the study results. This step opened on September 16, 2025, and closed on October 9, 2025. To determine which options children would rank, the research team first consulted the PPI groups to ensure the choices were appropriate and gather additional suggestions.

Additionally, this survey repeated the 3 questions from the Pre-Trial Quiz to assess children’s understanding of RCTs and learning gained through participation. Finally, children were asked about their experience of taking part in The Kid’s Trial and their views on the learning materials. This survey is available in Multimedia Appendix 6.

## Results

### Participation and Demographics

**Figure 4.**
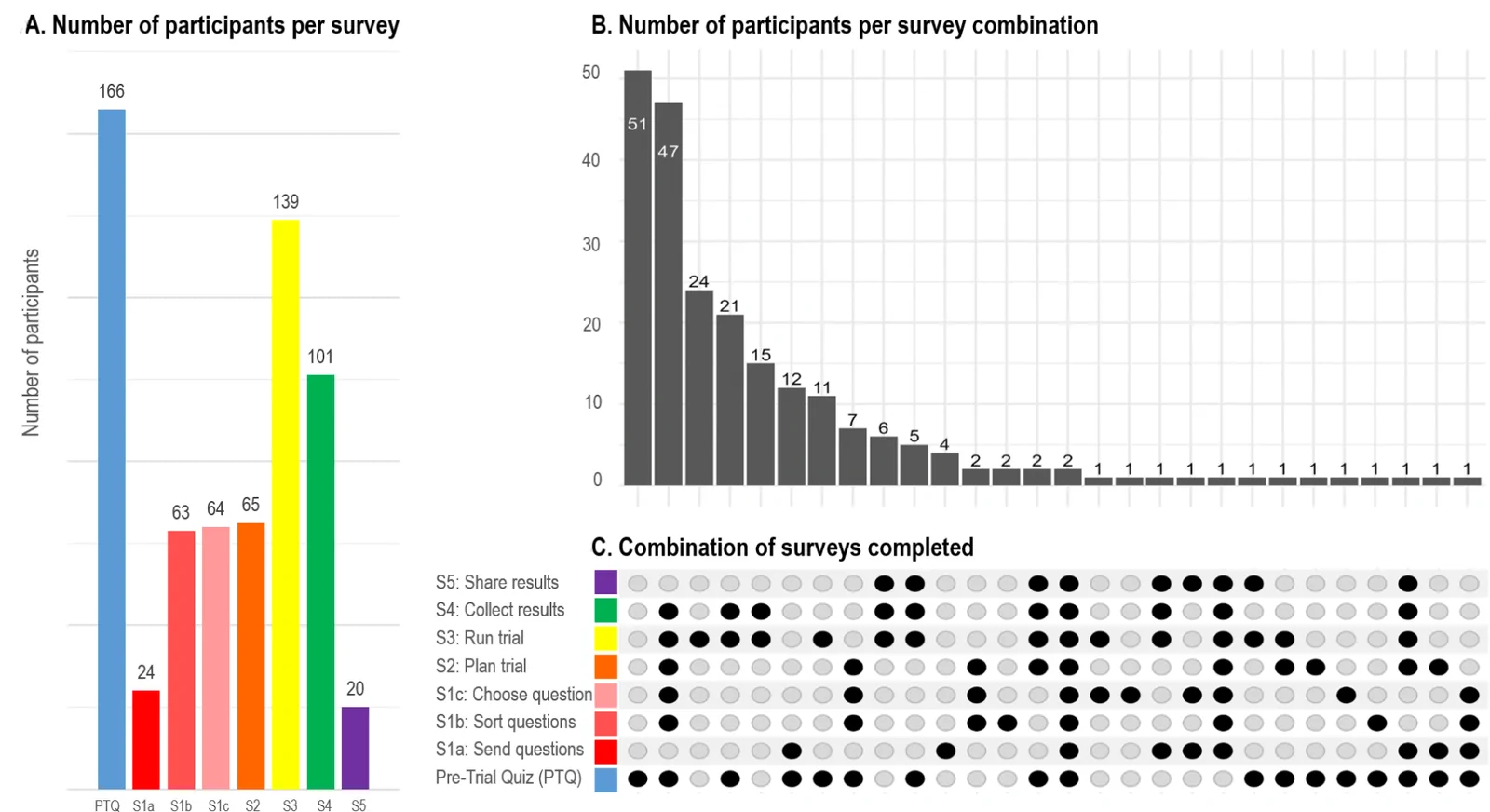
Overall participation across surveys and the combinations of surveys participants completed.A) Number of participants completing each survey. (B) Number of participants completing each combination of surveys. (C) Surveys included in each completion combination.

Participation across the steps of The Kid’s Trial is summarized in Figure 4. In total, 224 children from 15 countries took part in at least one survey (Figure 4A). The number of participants per survey ranged from 20 to 166. The optional Pre-Trial Quiz had the highest participation (n=166), followed by “Running the trial!” (n=139) and “Collecting the results!” (n=101). The earlier surveys (“Sending in your questions!” “Sorting the questions!” “Choosing the question!” and “Planning the trial!”) had lower participation counts (24, 63, 64, and 65 participants, respectively).

Figure 4B and C illustrate how participation overlapped across steps. Figure 4B shows the number of children who completed each unique combination of surveys, ordered from the most common (left) to the least common (right). Each bar represents a specific participation pattern. Figure 4C visualizes these combinations: filled circles indicate the surveys completed for each pattern, while empty circles show surveys not completed. Together, these plots reveal that 37% (n=82) completed only one survey, 19% (n=42) completed two, 15% (n=33) completed three, 6% (n=14) completed four, 0.9% (n=2) completed five surveys, and 21% (n=48) completed six surveys. Only 0.4% (n=1) completed seven surveys, and 0.9% (n=2) completed all eight. The most common participation patterns involved combinations of the trial-conducting steps (“Running the trial!” and “Collecting the results!) with or without the Pre-Trial Quiz.

The mean age of participants was 9.3 (SD 1.6) years. Most parents reported their child’s gender; 55% (n=122) listed “boy,” 45% (n=101) listed “girl,” 0.4% (n=1) selected “prefer not to answer,” and no parents selected “something not listed here.” Most parents provided their child’s country of residence (99%, n=222) and ethnicity (98%, n=219). Of those, approximately 10% (n=20) chose to specify ethnicity in a free-text response. Most participants lived in Ireland (44%, n=98) or Kenya (39%, n=87). Most parents listed “White” (53%, n=116) as their child’s ethnicity, and 34% (n=75) listed “Black/Black Irish.” Detailed demographic information of participating children is presented in Table 1.

**Table 1.** Demographics of all participants in The Kid’s Trial. Percentages use nonmissing responses as the denominator for each characteristic.

Characteristic (N=224)	Values
Age (years), n (%)	224 (100)
7	38 (17.0)
8	33 (14.7)
9	53 (23.7)
10	42 (18.3)
11	36 (16.1)
12	22 (9.8)
Gender, n (%)	224 (100)
Boy	122 (54.5)
Girl	101 (45.1)
Prefer not to answer	1 (0.4)
Country of residence	222 (100)
Ireland	98 (44.1)
Kenya	87 (39.2)
United Kingdom	15 (6.8)
United States	5 (2.3)
Australia	3 (1.4)
Germany	3 (1.4)
Estonia	2 (0.9)
India	2 (0.9)
Austria	1 (0.5)
Italy	1 (0.5)
Mexico	1 (0.5)
Northern Ireland	1 (0.5)
Pakistan	1 (0.5)
Singapore	1 (0.5)
Switzerland	1 (0.5)
Ethnicity, n (%)	219 (100)
White	116 (53.0)
Black/Black Irish	75 (34.2)
Other/Mixed	21 (9.6)
Asian/Asian Irish	7 (3.2)
Specific ethnic group, n (%)	170 (100)
Irish	68 (40.0)
African	66 (38.8)
White other	15 (8.8)
Mixed	11 (6.5)
Other	4 (2.4)
Asian other	2 (1.2)
Indian/Pakistani/Bangladeshi	2 (1.2)
Chinese	1 (0.6)
Traveler	1 (0.6)
Ethnicity free-text (n)	20 (100)
Asian Irish	2 (10)
Luo	2 (10)
White and Asian	2 (10)
American	1 (5)
British	1 (5)
Canadian Irish	1 (5)
Half British Australian/half Romanian	1 (5)
Indian	1 (5)
Indonesian	1 (5)
Kenyan	1 (5)
Kikuyu	1 (5)
Mexicano	1 (5)
Slovak and Latin American	1 (5)
Sri Lankan	1 (5)
White and Black	1 (5)
White and Black Caribbean	1 (5)
White and Eastern European	1 (5)

### Recruitment

#### Overview

**Figure 5.**
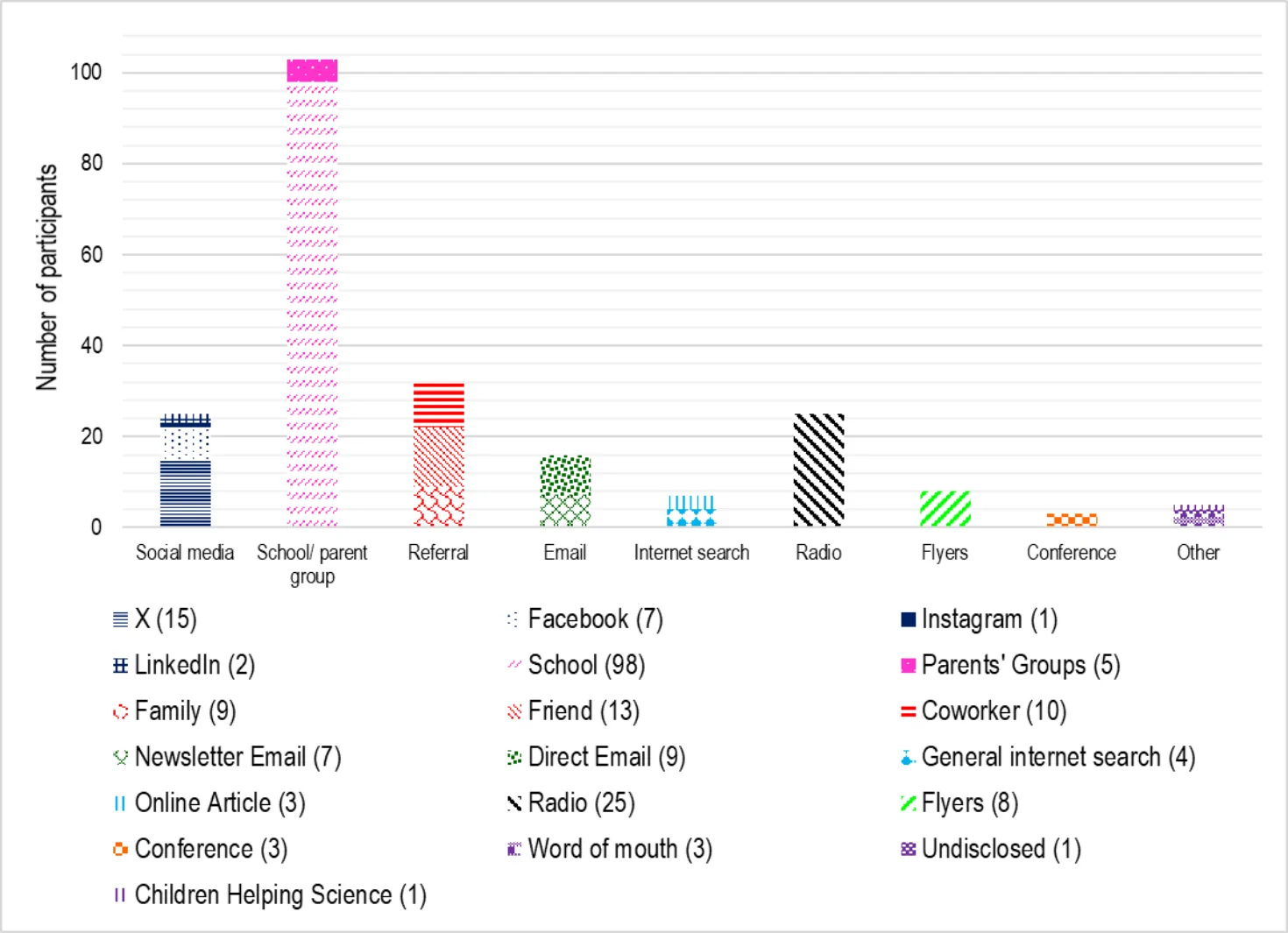
Recruitment sources for The Kid’s Trial.

Recruitment sources for The Kid’s Trial are shown in Figure 5. Of the 224 participants, the largest number was recruited through schools (44%, n=98), either via flyers circulated in Irish schools or through a Kenyan children’s charity, Toto Centre Initiative, which shared information about the study with local schools. Referrals from family, friends, or coworkers (14%, n=32) and social media or radio interviews (11%, n=25, each) were the next most effective recruitment avenues. Paid advertising increased website traffic but did not translate into participant enrollment.

#### Pre-Trial Quiz

Across the course of The Kid’s Trial, 166 children completed the Pre-Trial Quiz. This likely reflects that the survey remained open throughout The Kid’s Trial (Figure 2) to capture baseline knowledge of RCTs from participants joining at different stages of the study. The majority of children who completed this survey felt they understood what randomized trials were (70%, n=117), why they mattered (72%, n=120), and how they were conducted (60%, n=99).

#### Choosing the Question: Children Chose the Trial Question

In the first part of the question identification process, 24 children submitted questions they wanted to test in an RCT. After applying the inclusion criteria and PPI review, 9 questions were considered candidate questions for The Kid’s Trial. The full list of questions, along with the reasons for inclusion or exclusion, was published on the website and can be found in Multimedia Appendix 7.

**Figure 6.**
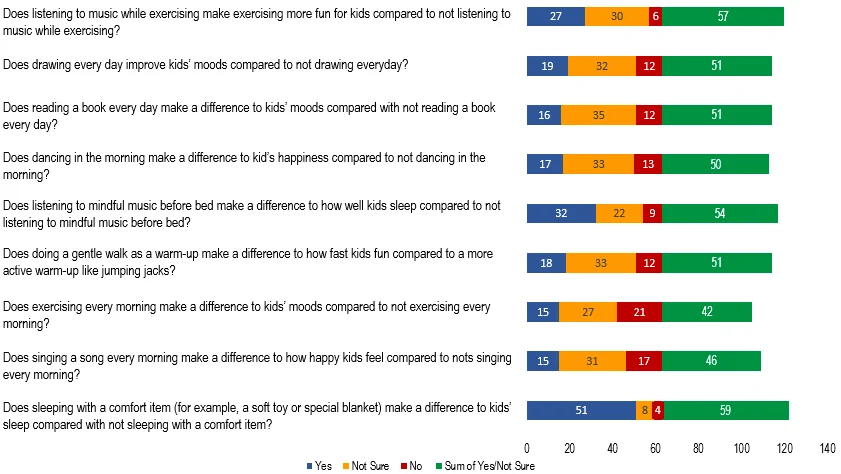
Results of “Sorting the questions!”

In “Sorting the questions!” 63 children reviewed the 9 candidate questions by completing a survey that used a 3-point Likert scale illustrated with emoji faces and corresponding text labels: “Yes, please. I’m interested in answering this question,” “I’m not sure about this question,” and “No, I’m not interested in answering this question.” The “Yes…” and “I’m not sure…” votes were combined to determine the top 3 questions. Figure 6 presents the results of this survey, in which “Does sleeping with a comfort item (for example, a soft toy or special blanket) make a difference to kids’ sleep?” (n=59 summed votes), “Does listening to music while exercising make exercising more fun for kids compared to not listening to music while exercising?” (n=57 summed votes), and “Does listening to mindful music before bed make a difference to how well kids sleep compared to not listening to mindful music before bed?” (n=54 summed votes) were the 3 highest-ranked questions.

In “Choosing the question!” 64 children ranked the top 3 questions and 73% (n=47) selected “Does sleeping with a comfort item (for example, a soft toy or special blanket) make a difference to children’s sleep compared with not sleeping with a comfort item?” as their favorite question.

#### Planning the Trial: Children Co-Designed the Trial

Sixty-five children took part in this survey to choose the outcomes of the trial and parameters of the “Try-it-Out” and “Wait-and-See” groups. Children decided that participants in the “Try-it-Out” group would:

1. Sleep with the same comfort item each night of the trial.
2. Start using their comfort item when they are getting ready for bed each night of the trial.
3. Sleep in their “usual” bed for the duration of the trial.
4. Keep everything else the same as normal in their bedtime routine each night of the trial.

The participants in the “Wait-and-See” group would:

1. Not use any comfort item each night.
2. Sleep in their “usual” bed each night.
3. Keep everything else in their bedtime routine the same as normal each night.

Children selected daytime sleepiness (40%, n=26) as the primary outcome and overall sleep quality (32%, n=21) as the secondary outcome. The full results of this survey are available in Multimedia Appendix 8.

#### Running the Trial: Who Joined the REST Trial?

The REST trial aimed to recruit 292 children to determine whether sleeping with a toy or comfort item affects children’s sleep compared with not sleeping with one. Details of how we calculated the sample size are available in the SAP [32] and the trial protocol [31]. Despite recruitment strategies and extending the planned 6-month recruitment period by 3 months, we were unable to reach this target. In total, 139 children from 11 countries were randomized (72 to the “Wait-and-See,” and 67 to the “Try-it-Out” group). Table 2 presents the baseline data for children enrolled in the REST trial. The mean age of participants was 9.8 (SD = 1.6) years, and 54% (n=76) were girls. At baseline, most children (80%, n=110) used a comfort item “Sometimes” or “Always.” At baseline, the overall mean SRI T-score was 51.38 (SD 7.94), and the overall mean SQS score was 3.76 (SD 0.81). These scores were similar across arms.

**Table 2.** Baseline data of children who joined the Randomized Evaluation of Sleeping with a Toy or comfort item (REST) trial. Percentages use nonmissing responses as denominators.

Characteristics	Variables		
	Overall (n=139)	Control (n=72)	Intervention (n=67)
Age (years), mean (SD)	9.8 (1.6)	9.8 (1.6)	9.8 (1.6)
Age (years), n (%)			
7	16 (11.5)	8 (11.1)	8 (11.9)
8	16 (11.5)	8 (11.1)	8 (11.9)
9	27 (19.4)	17 (23.6)	10 (14.9)
10	31 (22.3)	12 (16.7)	19 (28.4)
11	24 (17.3)	14 (19.4)	10 (14.9)
12	25 (18.0)	13 (18.1)	12 (17.9)
Gender, n (%)			
Boy	63 (45.3)	33 (45.8)	30 (44.8)
Girl	76 (54.7)	39 (54.2)	37 (55.2)
Something not listed here	—^a^	0 (0.0)	0 (0.0)
Prefer not to answer	—	0 (0.0)	0 (0.0)
Usual comfort-item use at baseline, n (%)			
Never	29 (20.9)	15 (20.8)	14 (20.9)
Sometimes	48 (34.5)	30 (41.7)	18 (26.9)
Always	62 (44.6)	27 (37.5)	35 (52.2)
Sleep-Related Impairment T-Score^b^, mean (SD)	51.4 (7.9)	51.2 (7.7)	51.5 (8.2)
Sleep quality, mean (SD)	3.8 (0.8)	3.8 (0.8)	3.7 (0.8)

a Not applicable.

b Sleep-Related Impairment T-Score (lower=less impairment).

#### Collecting the Results: Did Sleeping With a Comfort Item Affect Children’s Sleep?

**Figure 7.**
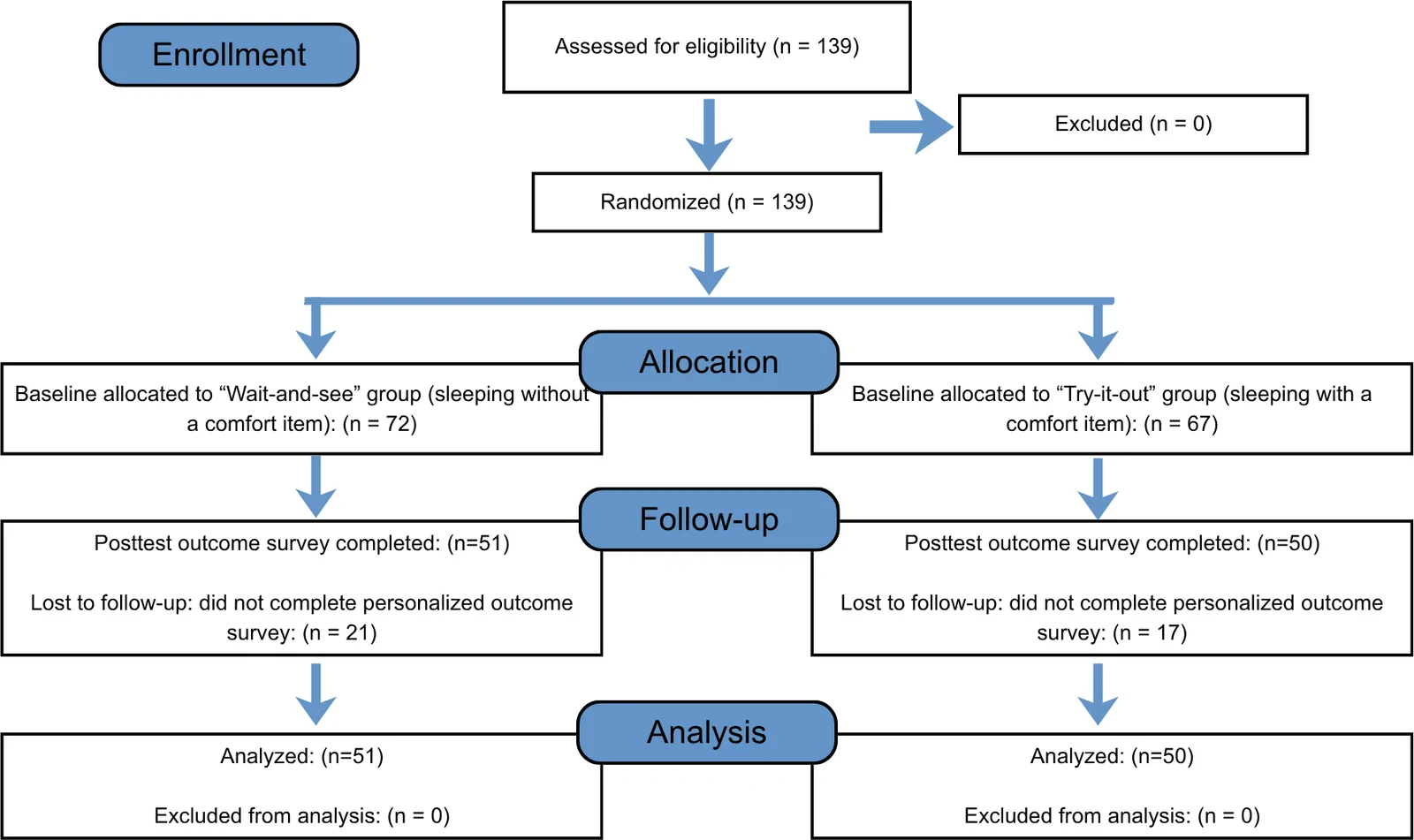
The Randomized Evaluation of Sleeping With a Toy or Comfort Item (REST) trial participant flow diagram.

Of the 139 children randomized in the REST trial, 101 completed their posttrial surveys, resulting in an attrition rate of approximately 27%. Figure 7 shows the CONSORT (Consolidated Standards of Reporting Trials) [33] participant flow diagram of the REST trial.

Adjusting for baseline outcome, baseline comfort-item use, age, and gender as fixed effects and country of residence as a random effect, the mean difference in SRI T-scores was −0.53 (95% CI −3.40 to 2.34; *P*=.71), indicating no meaningful effect on sleep-related impairment. The adjusted mean difference in SQS was 0.28 (95% CI 0.01-0.55; *P*=.04), representing a small, uncertain improvement in perceived sleep quality that was not maintained in per-protocol analyses. Multiple imputation and sensitivity analyses for the primary and secondary outcomes yielded consistent conclusions. No adverse events were reported. The primary analysis results from the REST trial are presented in Table 3. Full details of the trial’s methods and results are available elsewhere [30].

**Table 3.** Results of the primary analysis of the primary and secondary outcomes of the Randomized Evaluation of Sleeping With a Toy or Comfort Item (REST) trial. Models adjust for baseline outcome, baseline comfort-item use, age, and gender;, with random intercept for collapsed country.

POutcome and grouprimary analysis (ITT): adjusted means at follow-up and adjusted differences		Primary analysis (ITT):^a^ adjusted means at follow-up and adjusted differences	
Outcome and group		Adjusted mean (95% CI)	*P* value
SRI^b^ (lower=better**)**			
Control		47.73 (45.77 to 49.68)	
Intervention		47.20 (45.10 to 49.30)	
Difference (Intervention – Control)		−0.53 (−3.4 to 2.34)	.71
SQS^c^ (higher=better)			
Control		3.71 (3.52 to 3.89)	
Intervention		3.99 (3.79 to 4.19)	
Difference (intervention – control)		0.28 (0.01 to 0.55)	.04

a ITT: intention-to-treat. All randomized participants with posttest outcomes.

b SRI: sleep-related impairment . Lower=less impairment (better).

c SQS: sleep quality scale. Higher=better sleep quality.

#### Sharing the Results: Children Choose How to Share the Results of the Kid’s Trial

Of the 224 children invited, 20 children (9%) completed this survey. The 2 most popular ways to share the results of The Kid’s Trial were an animated video (50%, n=10) and charts and graphs (30%, n=6). The most popular places to share the results were The Kid’s Trial website (60%, n=12) and The Kid’s Trial YouTube channel (20%, n=4).

This survey also asked children the same questions that the Pre-Trial Quiz asked about their knowledge of RCTs. Of the 20 children who completed the final survey, 11 also completed the Pre-Trial Quiz. Their wider participation patterns are shown in Figure 4. Because posttrial survey completion was low, the data were insufficient for meaningful comparison with pretrial responses, and no statistical analyses were undertaken. However, of the 20 children who did answer these questions, all participants said they understood what randomized trials were and how they were conducted, and 90% (n=18) said they understood why they mattered.

Finally, we asked how children viewed participating in The Kid’s Trial and the materials on the website. Most children enjoyed taking part (95%, n=19), found the videos (85%, n=17) and written information (70%, n=14) on the website helpful, and said their interest in health research was increased (75%, n=15) due to taking part in The Kid’s Trial. The full results of this survey are available in S9 in Multimedia Appendix 9.

## Discussion

### Principal Findings

The Kid’s Trial demonstrated that a global, child-led citizen science RCT can be conducted using a fully decentralized design. Children engaged across multiple stages of the research process, including choosing a research question, defining trial procedures, participating in the trial, and choosing how the findings would be shared. While recruitment for the REST trial fell short of its target and attrition was higher than expected, the successful completion of all study steps and overall engagement of 224 children and families demonstrates that children can meaningfully contribute to core trial activities within a fully online RCT. These findings highlight key methodological and practical considerations for designing and conducting participatory, decentralized trials with children.

The embedded REST trial found no meaningful effect of comfort items on sleep-related impairment, with a small improvement in perceived sleep quality that was not robust to sensitivity analyses. These findings should be interpreted in the context of an underpowered study. As such, the REST trial is best understood as a proof-of-concept that demonstrates the implementation of a child-led, decentralized RCT, rather than providing definitive evidence on the effect of comfort items on the quality of children’s sleep. While the trial findings were inconclusive, the successful conduct of a participatory, child-led trial, demonstrated by children’s participation across all steps, illustrates that children can meaningfully engage with the process of generating, refining, and testing research questions. It also highlights that participation in these processes can provide opportunities for children to learn an important principle: not all interventions show effects, and learning to interpret null or uncertain findings is an important aspect of critical appraisal.

By adapting the principles of public co-production for a younger audience, The Kid’s Trial extends the approach pioneered by The People’s Trial [9]. Like its predecessor, it used experiential participation to engage children with RCT processes, aligning with educational frameworks that emphasize active participation as central to learning [34]. Children’s engagement throughout the study supports previous research showing that inquiry-based participation fosters curiosity and critical thinking about science and health [12,35,36]. Importantly, the study also provided researchers with practical insight into how children can be active partners in health literacy and research engagement initiatives, rather than passive recipients of information. Involving children in the co-creation of the trial meant that they chose the research question, selected and prioritized outcomes, contributed to trial procedures, and selected dissemination routes according to their own priorities and perspectives. As a result, The Kid’s Trial reflects children’s interests and decision-making processes in ways that may have differed substantially from an adult-designed study.

One of the aims of The Kid’s Trial was to explore whether participation in a co-created RCT could improve children’s engagement with and understanding of trials. The limited posttrial results are consistent with respondents’ perceived improvements in RCT understanding. These findings should be interpreted cautiously, given the small number of posttrial responses and potential self-selection bias, but they are consistent with the broader educational rationale underpinning participatory approaches.

It is important, however, to distinguish between the dual objectives of The Kid’s Trial. While the study successfully engaged children in an experiential citizen-science initiative, the embedded REST trial fell short of its recruitment target, was underpowered for its primary analysis, and yielded null or uncertain findings. These dual outcomes underscore the tension in citizen science between rigorous scientific methods and broad public engagement. While this tension is not unique to our study, it highlights the importance of clearly defining success criteria at the outset: for educational citizen-science initiatives, engagement and learning outcomes may be more relevant benchmarks than statistical power or definitive scientific conclusions.

Participation patterns showed considerable variability but demonstrated that sustained engagement in a multistep online study is achievable for children in this age group. Because the final survey sample was small, we did not attempt demographic comparisons between early- and late-stage completers. While many participants engaged briefly, a meaningful subset remained active across several stages, particularly during the trial conduct steps. Children and parents appeared to be most motivated by concrete, action-oriented activities such as running the trial and reporting results, with fewer joining the earlier planning steps. This likely reflects a combination of factors, including the intensified recruitment efforts during that period and increased awareness of the study after several months of ongoing advertising and dissemination of materials. This pattern suggests future studies should consider a “core-periphery” design that distinguishes between essential trial-conduct steps (which attract higher engagement) and optional enrichment activities (for sustained participants). Alternatively, condensing all steps into a shorter, more intensive timeframe might preserve momentum, though this would need to be balanced against the cognitive and time demands on child participants.

Recruitment for The Kid’s Trial proved challenging despite extensive and varied outreach. The intentionally wide target population, chosen to maximize inclusivity, may have reduced the personal relevance or urgency that drives participation in more narrowly focused studies, particularly given the many competing demands on families’ schedules. Additionally, the lack of established school or community partner networks limited the reach of the recruitment effort. The REST trial’s extended recruitment period (9 vs 6 months) may have contributed to attrition, as delays between steps reduced continuity and momentum. The final step, “Sharing the results!” was open for only 2 weeks due to study deadlines, which may have further contributed to the low participation at that stage. The decline to only 9% completion in the final step raises important questions about study design. While the abbreviated timeframe was a contributing factor, this attrition likely also reflects cumulative fatigue, the absence of personal relevance of disseminating the outcome, and the substantial time elapsed since randomization (up to 9 months for early participants). Future studies might maintain engagement by integrating dissemination planning earlier, offering multiple dissemination formats throughout the study, or creating milestone celebrations to sustain momentum. The low response rate also prevented robust assessment of learning outcomes, limiting our ability to evaluate whether participation meaningfully improved trial literacy, a core objective of the initiative.

Notably, paid digital advertising increased website traffic but failed to convert visitors to participants, suggesting that trust and personal connection, rather than visibility alone, drive enrollment in child-focused citizen science. This finding has practical implications for future resource allocation, suggesting that investment in community partnerships and institutional networks may yield better returns than paid advertising.

Despite these challenges, the study demonstrates the feasibility of this approach. Early and sustained involvement of PPI groups ensured that study materials were age-appropriate and engaging. The decentralized model allowed participation across cultural and geographic contexts at relatively low cost, showing that such designs can enhance inclusion and accessibility in child-focused research.

### Strengths and Limitations

The Kid’s Trial had several notable strengths. It is, to our knowledge, the first global citizen-science RCT co-created and conducted by children, integrating authentic public involvement throughout the trial. The decentralized design allowed participation from 15 countries without prespecified geographic limitations. However, participation was heavily concentrated in 2 countries (Ireland and Kenya, representing 83% of participants), reflecting the research team’s networks and the successful partnership with Toto Centre Initiative in Kenya. While the study achieved international reach, future work should distinguish between “global access” and “global engagement” when describing decentralized studies. The extensive PPI input enhanced the study’s relevance and child-centered design, and the trial materials, including the animations, simplified complex trial concepts, making them accessible and engaging for children.

However, the limitations should also be acknowledged. The online format excludes children without internet access, estimated at up to one-third of the global population [37]. At the same time, the decentralized format may offer opportunities to engage children who are geographically dispersed or less connected to traditional research settings, particularly when combined with community-based partnerships. The reliance on parental support and the use of English-only materials also limited inclusivity. Although our work with the CRAG members enhanced the accessibility and child-friendly tone of study materials, the study did not address the needs of children with additional communication or learning requirements. In addition, we did not collect children’s literacy or parents’ education levels among PPI group members, which limits how fully we can describe the advisory groups’ educational and literacy context or assess whether further adaptations were needed. Future work should explore how participatory trials can be adapted to support a wider range of access needs, for example, through easy-read or multilingual materials, additional visual supports, hybrid delivery options, and accessibility testing with children at the younger end of the age range.

The REST trial was underpowered, and its outcomes and children’s understanding of RCTs was self-reported. Because participants were aware of their allocated group and outcomes were self-reported, the REST trial was also vulnerable to expectancy and social desirability biases. This may have influenced the outcome measures, such as perceived sleep quality, particularly given that the small between-group difference in SQS was not robust to sensitivity analyses. Posttrial data were limited to 20 responses (9% of participants), and although all respondents reported understanding what RCTs were and how they were conducted, compared with 70% and 60% at baseline, respectively, these findings require cautious interpretation, given the small sample size and potential selection bias (those who completed the final survey may have been more engaged learners). Rather than providing definitive evidence of learning, these results are consistent with the possibility that experiential participation may support children’s perceived understanding of trial concepts and their importance. Future studies should prioritize learning assessment by embedding brief knowledge checks throughout the study rather than relying solely on final surveys, and by using validated instruments where available.

### Conclusions

The Kid’s Trial provides proof-of-concept that children can meaningfully contribute to the design and conduct of an RCT through a fully decentralized participatory approach. It highlights both the potential and the practical challenges of involving children as research partners. By introducing trial literacy through experiential participation, the study suggests that children can begin to engage critically with evidence from an early age through active participation in generating and testing research questions.

Future child-led citizen-science studies could improve this model by prioritizing early partnerships with schools and community organizations, which proved the most effective recruitment channel in the present study (44% of participants), rather than relying on social media or paid advertising, which generated traffic but failed to convert visitors to participants. Condensing study phases to maintain momentum between steps (limiting intervals to 4‐6 weeks rather than the months-long gaps in our timeline) and integrating brief learning assessments throughout rather than only at the end point would address the substantial attrition observed in later stages. Researchers should also clearly distinguish between educational outcomes (engagement, interest, and satisfaction) and scientific outcomes (statistical power and definitive findings) when defining success metrics, as these dual objectives may require different evaluation frameworks and can be in tension. Finally, incorporating gamification elements, milestone celebrations, and regular progress updates may help sustain participant engagement across multiple study phases, while developing multilingual materials and hybrid delivery models would enhance true global inclusivity beyond simply enabling international access.

## Supplementary material

10.2196/89240 Multimedia Appendix 1 Pre-Trial Quiz.

10.2196/89240 Multimedia Appendix 2 Sending in your question! survey.

10.2196/89240 Multimedia Appendix 3 Sorting the questions! survey.

10.2196/89240 Multimedia Appendix 4 Choosing the question! survey.

10.2196/89240 Multimedia Appendix 5 Planning the trial! survey.

10.2196/89240 Multimedia Appendix 6 Running the trial! survey.

10.2196/89240 Multimedia Appendix 7 Sharing the results! survey.

10.2196/89240 Multimedia Appendix 8 Results of the Planning the trial! survey.

10.2196/89240 Multimedia Appendix 9 Results of the Sharing the results! survey.

## Abbreviations

CHS: Children Helping Science
CONSORT: Consolidated Standards of Reporting Trials
CRAG: Children’s Research Advisory Group
ECS: European Citizen Science
ITT: intention-to-treat
PPI: public and patient involvement
PRAG: Parents’ Research Advisory Group
PROMIS: Patient-Reported Outcomes Measurement Information System
RCT: randomized controlled trial
REST: Randomized Evaluation of Sleeping With a Toy or Comfort Item
SAP: statistical analysis plan
SQS: sleep quality scale
SRI: sleep-related impairment

## References

[1] Sibbald B, Roland M (1998). Understanding controlled trials. Why are randomised controlled trials important?. BMJ.

[2] Fogel DB (2018). Factors associated with clinical trials that fail and opportunities for improving the likelihood of success: a review. Contemp Clin Trials Commun.

[3] Pirosca S, Shiely F, Clarke M, Treweek S (2022). Tolerating bad health research: the continuing scandal. Trials.

[4] Natale P, Saglimbene V, Ruospo M (2021). Transparency, trust and minimizing burden to increase recruitment and retention in trials: a systematic review. J Clin Epidemiol.

[5] Yadav S, Todd A, Patel K (2022). Public knowledge and information sources for clinical trials among adults in the USA: evidence from a Health Information National Trends Survey in 2020. Clin Med (Lond).

[6] Langford A, Studts JL, Byrne MM (2021). Improving knowledge and decision readiness to participate in cancer clinical trials: effects of a plain language decision aid for minority cancer survivors. Patient Educ Couns.

[7] Mackenzie IS, Wei L, Rutherford D (2010). Promoting public awareness of randomised clinical trials using the media: the “Get Randomised” campaign. Br J Clin Pharmacol.

[8] (2025). Increasing clinical trial awareness. Clinical Trials For All.

[9] Finucane E, O’Brien A, Treweek S (2022). The people’s trial: supporting the public’s understanding of randomised trials. Trials.

[10] Mah VK, Ford-Jones EL (2012). Spotlight on middle childhood: rejuvenating the “forgotten years”. Paediatr Child Health.

[11] Shapiro T, Perry R (1976). Latency revisited. The age 7 plus or minus 1. Psychoanal Study Child.

[12] Nsangi A, Semakula D, Oxman AD (2020). Effects of the informed health choices primary school intervention on the ability of children in Uganda to assess the reliability of claims about treatment effects, 1-year follow-up: a cluster-randomised trial. Trials.

[13] (2021). Health literacy in the context of health, well-being and learning outcomes: the case of children and adolescents in schools. World Health Organization.

[14] Borges do Nascimento IJ, Pizarro AB, Almeida JM (2022). Infodemics and health misinformation: a systematic review of reviews. Bull World Health Organ.

[15] Hanley DF, Bernard GR, Wilkins CH (2023). Decentralized clinical trials in the trial innovation network: value, strategies, and lessons learned. J Clin Transl Sci.

[16] Price J, Goodson N, Warren EJ, Wicks P, Reites J (2021). Resilient design: decentralized trials recovered faster from the impact of COVID-19 than traditional site-based designs. Expert Rev Med Devices.

[17] Goodson N, Wicks P, Morgan J, Hashem L, Callinan S, Reites J (2022). Opportunities and counterintuitive challenges for decentralized clinical trials to broaden participant inclusion. NPJ Digit Med.

[18] Jean-Louis G, Seixas AA (2024). The value of decentralized clinical trials: inclusion, accessibility, and innovation. Science.

[19] Lepage S, Lepage L, Devane D (2025). Co-creating with the scientists of tomorrow: shaping trials and the future. The Kid’s Trial.

[20] Lepage S, Whelan B, Flight L, Totton N, Devane D (2025). Lessons learned from building the kid’s trial with an online children’s and parents’ research advisory group: a descriptive, qualitative study. Res Involv Engagem.

[21] (2025). AI-powered insights,backed by real experts. QuestionPro.

[22] (2025). Powtoon homepage. Powtoon Ltd.

[23] Lepage S (2025). The kid’s trial. YouTube.

[24] (2025). European citizen science platform. European Citizen Science Association.

[25] (2025). Children helping science homepage. Children Helping Science.

[26] Bevans KB, Meltzer LJ, De La Motte A, Kratchman A, Viél D, Forrest CB (2019). Qualitative development and content validation of the PROMIS pediatric sleep health items. Behav Sleep Med.

[27] (2022). PROMIS sleep scoring manual. Health Measures.

[28] Carle AC, Bevans KB, Tucker CA, Forrest CB (2021). Using nationally representative percentiles to interpret PROMIS pediatric measures. Qual Life Res.

[29] Snyder E, Cai B, DeMuro C, Morrison MF, Ball W (2018). A new single-item sleep quality scale: results of psychometric evaluation in patients with chronic primary insomnia and depression. J Clin Sleep Med.

[30] Lepage S, Flight L, Totton N, Devane D (2026). The REST (randomised evaluation of sleeping with a toy or comfort item) trial: an online, randomised trial of comfort item use on sleep quality in children. medRxiv.

[31] Lepage S, Flight L, Totton N, Devane D (2025). The REST (randomised evaluation of sleeping with a toy or comfort item) trial: a protocol for an online, randomised trial of comfort item use on sleep quality in children. Contemp Clin Trials Commun.

[32] Lepage S, Flight L, Totton N, Devane D (2025). The REST (randomised evaluation of sleeping with a toy or comfort item) trial OSF repository. Open Science Framework.

[33] Hopewell S, Chan AW, Collins GS (2025). CONSORT 2025 statement: updated guideline for reporting randomised trials. BMJ.

[34] Kolb D (1984). Experiential Learning: Experience as the Source of Learning and Development.

[35] Biesty L, Galvin S, Finucane E, Healy P, Devane D, Conway T (2020). Can learning about trials be child’s play? A qualitative exploration of the “Schools Teaching Awareness of Randomised Trials” (START) initiative. Trials.

[36] Blackawton PS, Airzee S, Allen A (2011). Blackawton bees. Biol Lett.

[37] (2024). Union measuring digital development: facts and figures 2024. International Telecommunication Union.

