# Supplementary material for: Feasibility and Methodological Reflections From the Co-Creation of a Randomized Controlled Trial (The Kid’s Trial): Decentralized, Child-Led Citizen-Science Study
Source: J Med Internet Res. 2026 Aug 28;28:e89240. doi: 10.2196/89240 (PMC13524311; doi:10.2196/89240)
Supplement: Supplementary file 1 [file jmir-v28-e89240-s008.pdf]

S8: Results of the 'Planning the trial!' survey

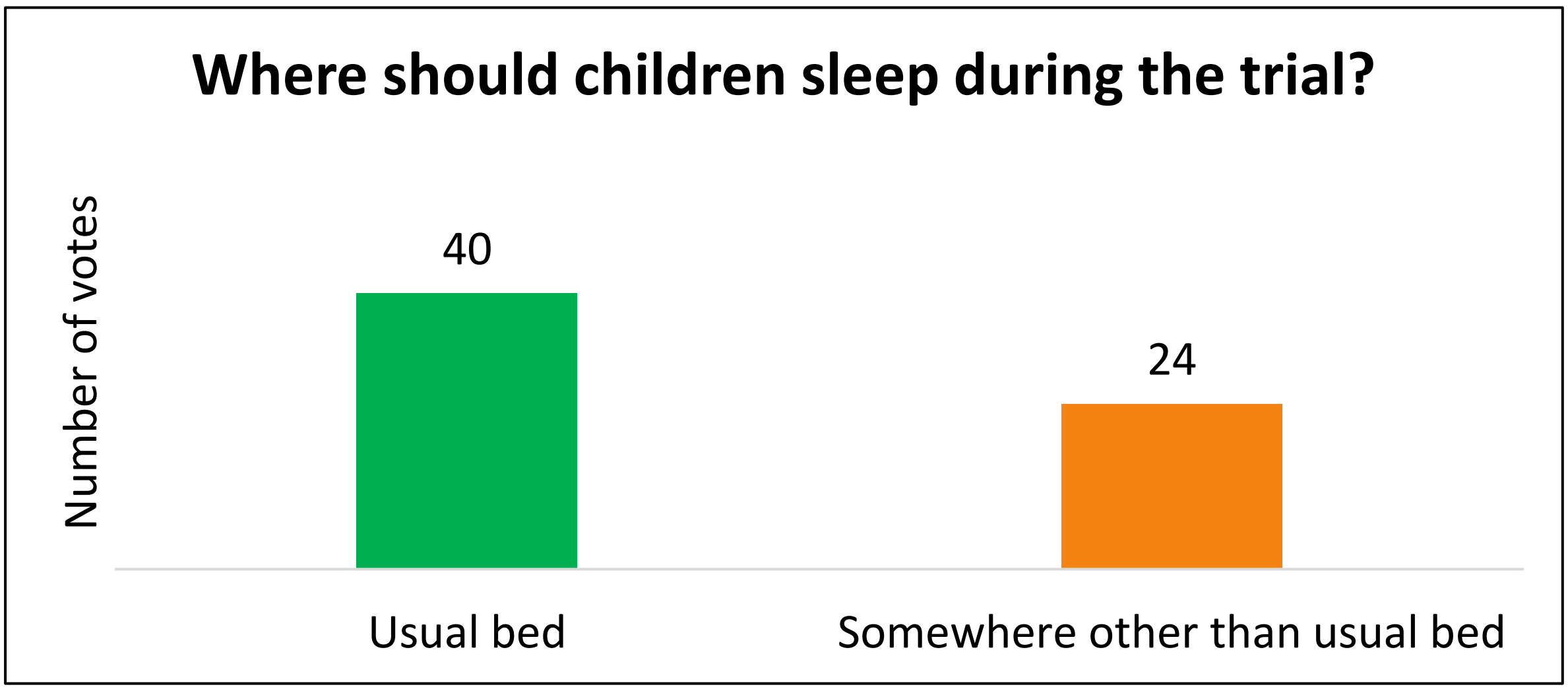

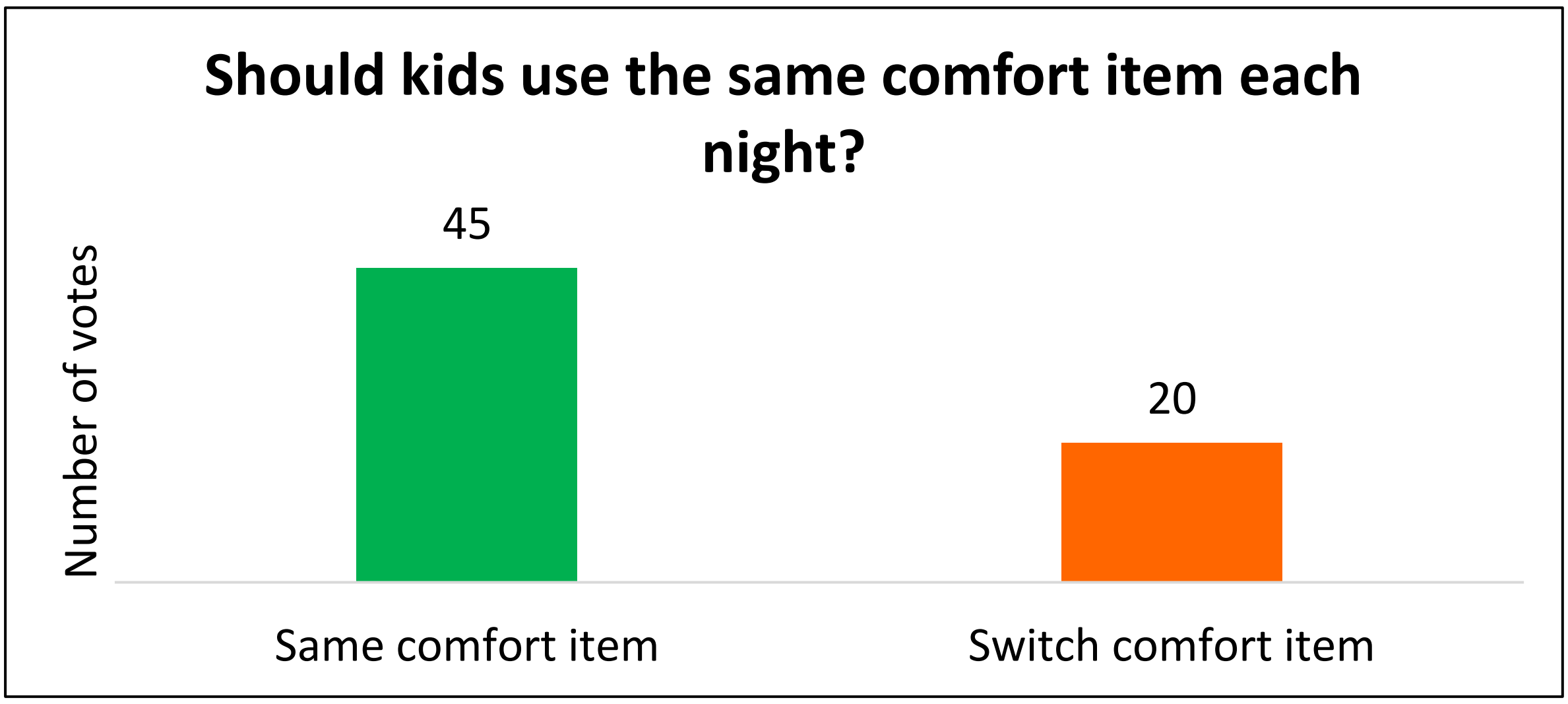

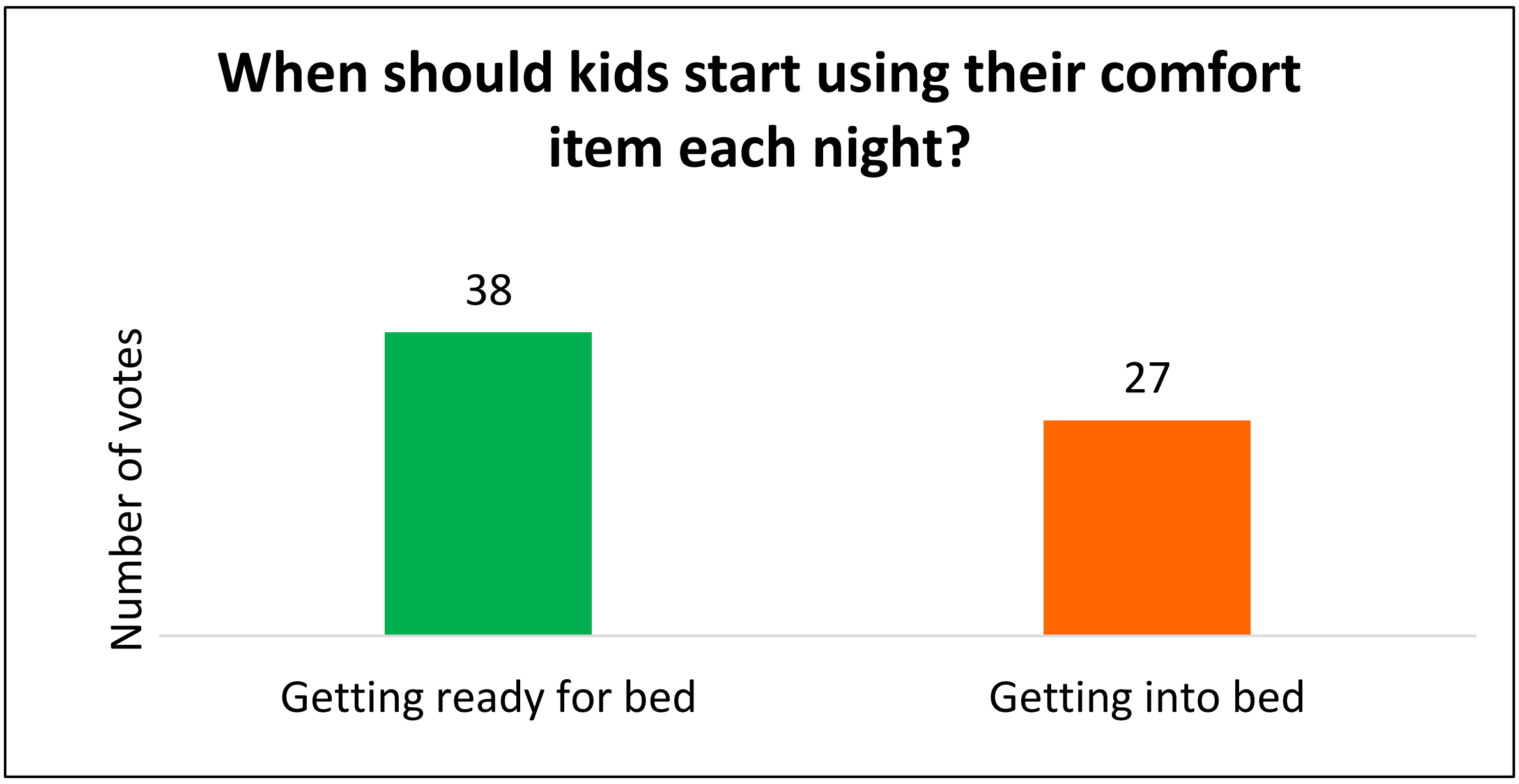

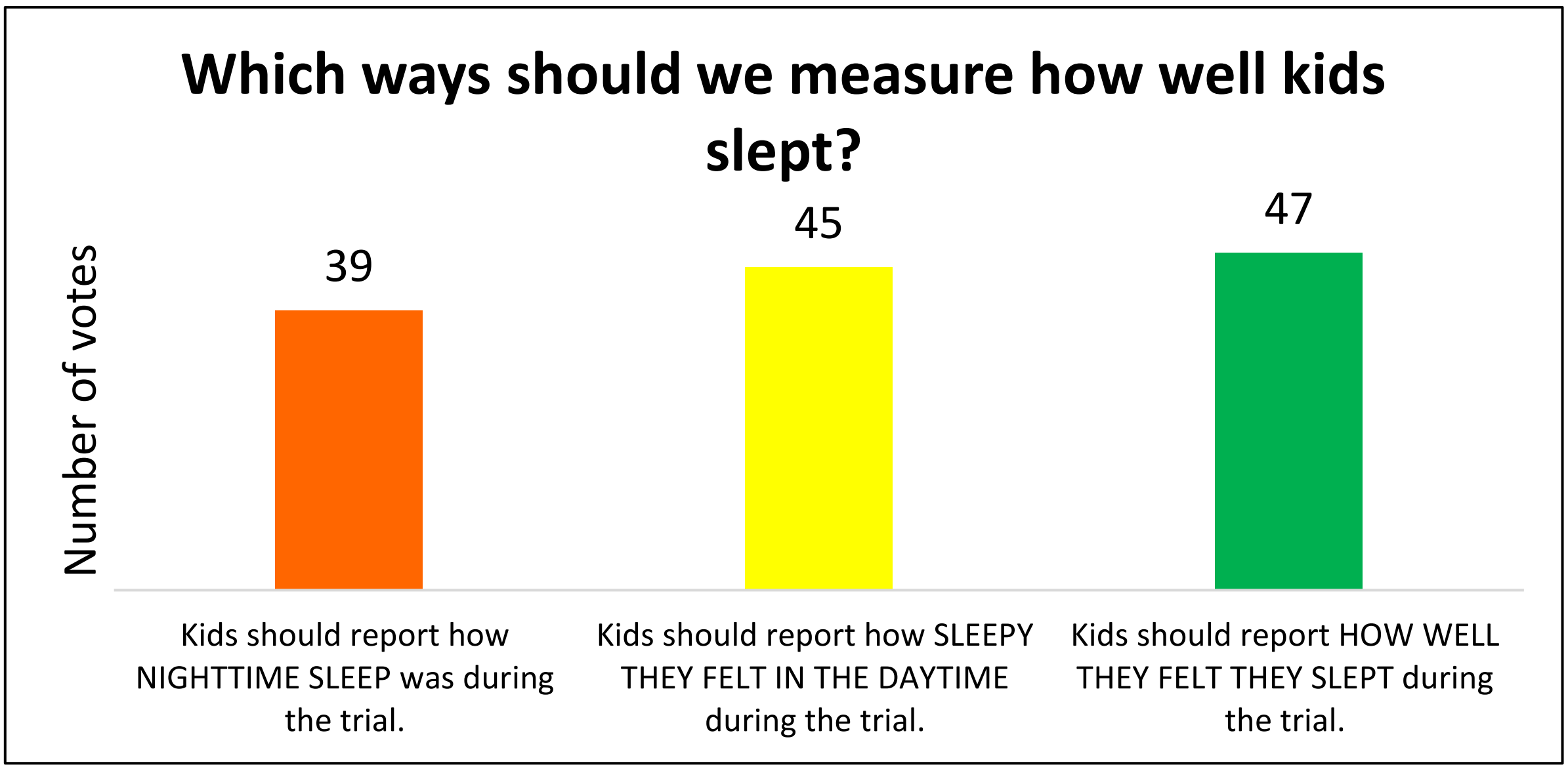

Which ways should we measure how well kids slept?
Number of votes
39
45
47
Kids should report how NIGHTTIME SLEEP was during the trial.
Kids should report how SLEEPY THEY FELT IN THE DAYTIME during the trial.
Kids should report HOW WELL THEY FELT THEY SLEPT during the trial.

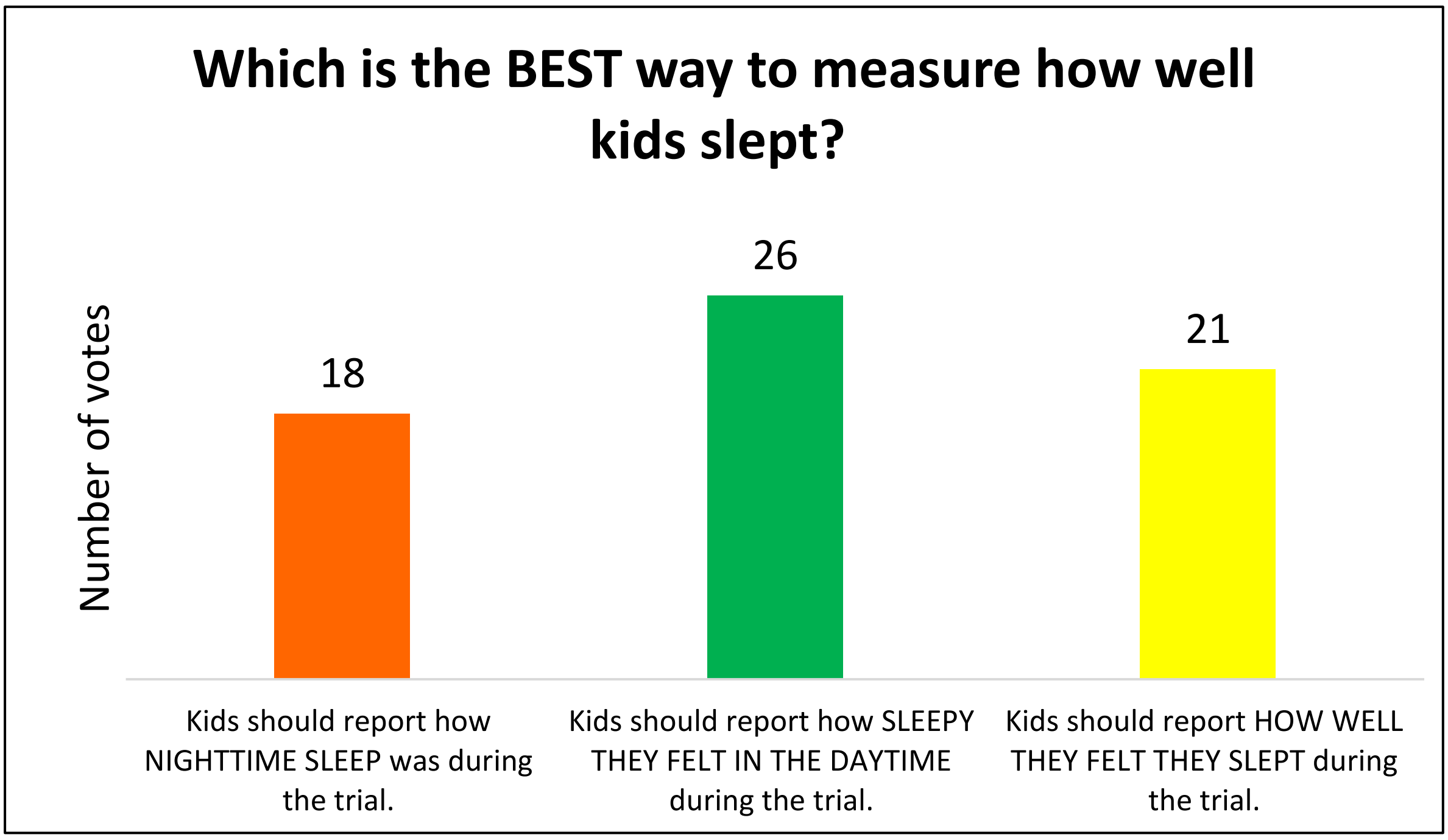

Which is the BEST way to measure how well kids slept?
Number of votes
18
26
21
Kids should report how NIGHTTIME SLEEP was during the trial.
Kids should report how SLEEPY THEY FELT IN THE DAYTIME during the trial.
Kids should report HOW WELL THEY FELT THEY SLEPT during the trial.
