# Supplementary material for: Feasibility and Methodological Reflections From the Co-Creation of a Randomized Controlled Trial (The Kid’s Trial): Decentralized, Child-Led Citizen-Science Study
Source: J Med Internet Res. 2026 Aug 28;28:e89240. doi: 10.2196/89240 (PMC13524311; doi:10.2196/89240)
Supplement: Supplementary file 2 [file jmir-v28-e89240-s009.pdf]

Supplemental Fig. S9: Results of the ‘Sharing the results!’ survey.

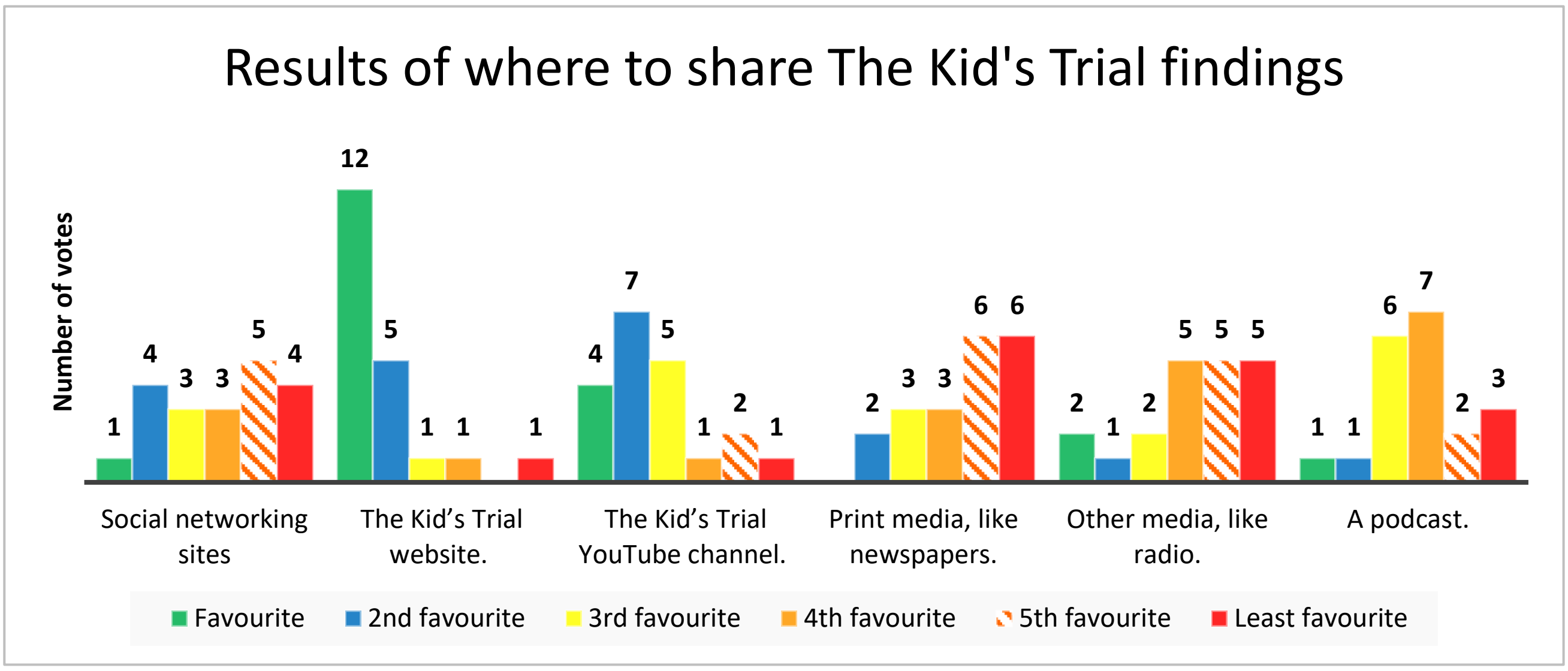

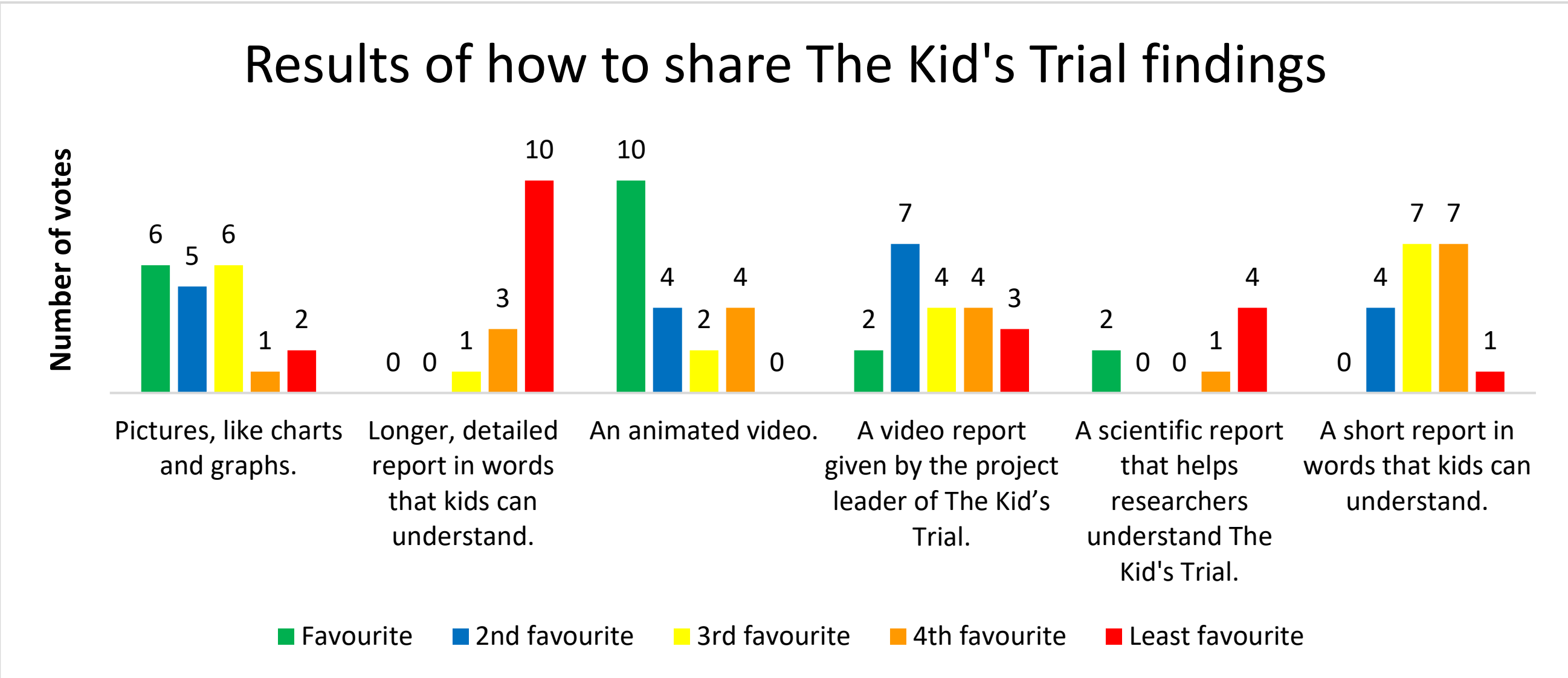

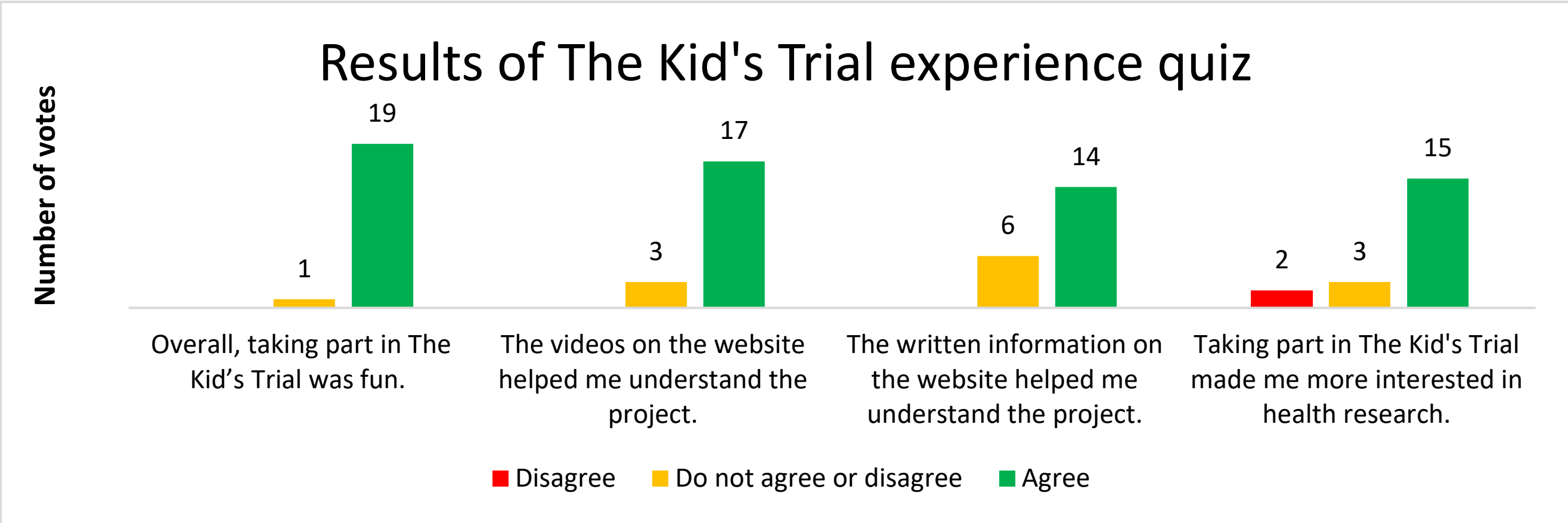
Results of The Kid's Trial experience quiz
Number of votes
19
1
17
3
14
6
15
2
3
Overall, taking part in The Kid's Trial was fun.
The videos on the website helped me understand the project.
The written information on the website helped me understand the project.
Taking part in The Kid's Trial made me more interested in health research.
Disagree
Do not agree or disagree
Agree

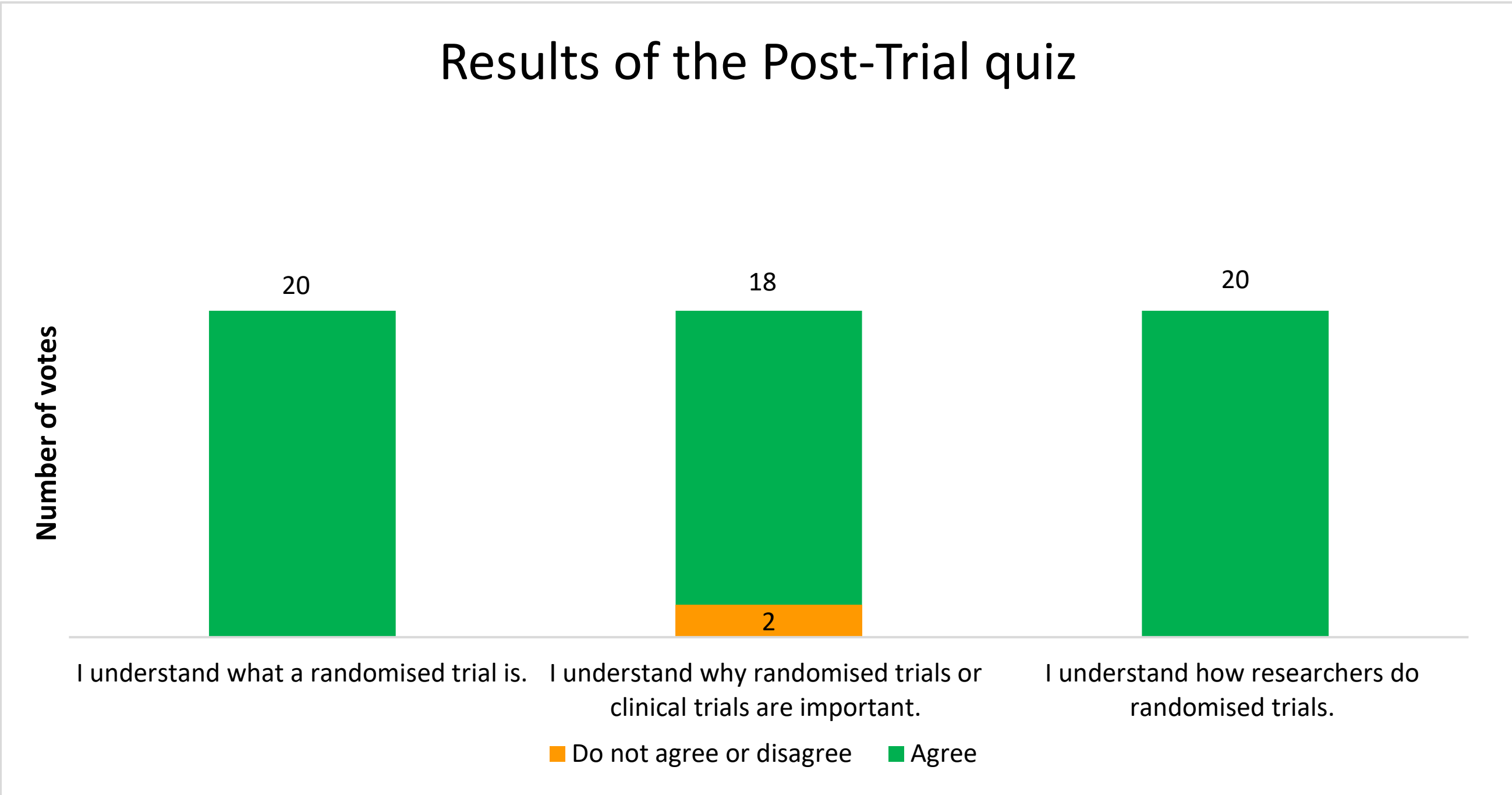
Results of the Post-Trial quiz
Number of votes
20
18
2
20
I understand what a randomised trial is.
I understand why randomised trials or clinical trials are important.
I understand how researchers do randomised trials.
Do not agree or disagree
Agree
