# Supplementary material for: Feasibility and Methodological Reflections From the Co-Creation of a Randomized Controlled Trial (The Kid’s Trial): Decentralized, Child-Led Citizen-Science Study
Source: J Med Internet Res. 2026 Aug 28;28:e89240. doi: 10.2196/89240 (PMC13524311; doi:10.2196/89240)
Supplement: Supplementary file 4 [file jmir-v28-e89240-s002.docx]

# Sending in your question!

## Step 1a

CONSENT FORM FOR PARENTS/CAREGIVERS/GUARDIANS

Research study title: The Kids Trial: An online randomised trial.

If you're happy for your child to take part in The Kid's Trial, please fill in this form and help your child fill in their own ASSENT form, too.

Remember, you must fill out a separate consent form for each child in your home who wants to join the trial. Please use a different email address for each child.

Please [make sure you have read or listened to the Parents' & Caregivers' Information Flipbook on the website.](https://thekidstrial.ie/)

- **Please read the following statements:**
  - **I have reviewed the** [**Parents' & Caregivers' Information Flipbook**](https://thekidstrial.ie/) **and the** [**Privacy Notice**](https://www.thekidstrial.ie/privacynotice) **on the website.**
  - **I can withdraw my child from The Kid's Trial at any time without giving any reason.**
  - **I have had an opportunity to think about the information in the Flipbook and Privacy Notice and had any questions answered by the Research Team.**
  - **I understand my child’s and my own rights in relation to this study.**
  - **I understand the data collected during this study will be processed in compliance with data protection laws as explained in the Parents’/Caregivers’ Information Flipbook and Privacy Notice on The Kid's Trial website.**
  - **I understand the data collected during this study will be securely stored for seven (7) years after the completion of the trial.**
  - **I understand that all information I provide will be kept confidential, subject to legal limitations. This means that if my child or I tell the researcher that someone is in immediate danger or risk of harm, the researcher is required to report this to the relevant authorities.**

**Yes, I understand and agree to the above statements.**

**No, I do not agree to the above statements.**

- **Consent**

Please answer the following:

**Yes	No**

**I am the legal guardian for the child for whom I am giving consent.**

**I agree that my child can join The Kid's Trial.**

Parent/Guardian Contact Information

**Parent/Guardian Full Name**

**Participating Child's Full Name**

**Parent/Guardian Email Address**

Please tell us a bit about your child.

- **My child is:**

**7 years old**

**8 years old**

**9 years old**

**10 years old**

**11 years old**

**12 years old**

- **What best describes your child's gender?**

**A boy****A girl**

**Something not listed here****Prefer not to answer**

In which country does your child live?

What best describes your child's cultural or ethnic background? This means ‘with what group do they share a background, language, culture or ancestry?

Please select an option from the drop down menu below. **After selecting an option, another drop down list will be displayed below.** Select an appropriate option from that list as well. **If you choose to write in a description, you may do so in the next question.**

-- Select --

If you choose to write in your child's ethnicity description, please do so here.

- **How likely would you be to let your child take part in a randomised or clinical trial if you were asked?**

**Extremely likely****Somewhat likely****Not sure**

**Somewhat unlikely****Extremely unlikely**

- **How did you hear about The Kid's Trial?**

Please select an option from the drop down menu below. **After selecting an option, another drop down list will be displayed below.** Select an appropriate option from that list as well. **If you choose to write in a description, you may do in the next question.**

-- Select --

You can tell us more about how you heard about The Kid's Trial here.

Please have your child fill out the next sections. You may help if you wish.

FOR THE KIDS

Both you and your parent or caregiver must agree to take part in The Kid's Trial. If you're both ready, please fill out the form.

- **Please read the sentences below and check the box to tell us you understand what The Kid’s Trial is and what we are doing. You can** [**find all this information on The Kid's Trial website in the Kids' Information Flipbook.**](https://www.thekidstrial.ie/)
  - **I have read or listened to the** [**Kid's Information Flipbook**](https://www.thekidstrial.ie/) **(or had my parent/caregiver explain it to me) on The Kid's Trial website.**
  - **I can stop being part of The Kid's Trial at any time and no one will be upset.**
  - **I understand I will be asked to share my thoughts and opinions when I participate in The Kid's Trial.**
  - **I have talked about joining The Kid's Trial with my parent or caregiver and we have had our questions (if you had any) answered.**
  - **I understand that I (or my parent/caregiver) can contact the research team at any time if we have worries or questions about The Kid's Trial.**

**Yes, I understand what the sentences above say, and I agree with them.****No, I do not agree with the above sentences.**

- **I would like to join The Kid's Trial.**

**Yes****No**

It’s time for THE KIDS to tell us their questions for The Kid’s Trial!

You can use any of these examples or come up with your own question that you're curious about!

Here are some example questions:

I would like to find out if listening to music in the morning improves kids' moods compared to not listening to music in the morning.

I would like to find out if sleeping with a window open improves kids' sleep compared to not sleeping with a window open.

I would like to find out if doing jumping jacks before school improves kids' listening in class compared to not doing jumping jacks in the morning.

The things you need to think about are:

Is this a question we can find an answer to in about a week? Is this a question most kids could try and answer at home or somewhere close to where they live?

Is this a safe question to try and answer?

- **I would like to find out if (the thing you'd like us to try out, for example, listening to music in the morning):**
- **makes a difference to (what you want to change, for example, kids' moods):**
- **compared to ( the thing you'd like to compare, for example, not listening to music in the morning):**
