# Supplementary material for: Feasibility and Methodological Reflections From the Co-Creation of a Randomized Controlled Trial (The Kid’s Trial): Decentralized, Child-Led Citizen-Science Study
Source: J Med Internet Res. 2026 Aug 28;28:e89240. doi: 10.2196/89240 (PMC13524311; doi:10.2196/89240)
Supplement: Supplementary file 7 [file jmir-v28-e89240-s005.docx]

# Planning the trial!

### Step 2

**CONSENT FORM FOR PARENTS/CAREGIVERS/GUARDIANS**

**Research study title: The Kids Trial: An online randomised trial.**

**If you're happy for your child to take part in The Kid's Trial, please fill in this form and help your child fill in their own ASSENT form, too.**

**Remember, you must fill out a separate consent form for each child in your home who wants to join the trial. Please use a different email address for each child.**

**Please** [**make sure you have read or listened to the Parents' & Caregivers' Information Flipbook on the website.**](https://thekidstrial.ie/)

- **Please read the following statements:**
  - **I have reviewed the** [**Parents' & Caregivers' Information Flipbook**](https://thekidstrial.ie/) **and the** [**Privacy Notice**](https://www.thekidstrial.ie/privacynotice) **on the website.**
  - **I can withdraw my child from The Kid's Trial at any time without giving any reason.**
  - **I have had an opportunity to think about the information in the Flipbook and Privacy Notice and had any questions answered by the Research Team.**
  - **I understand my child’s and my own rights in relation to this study.**
  - **I understand the data collected during this study will be processed in compliance with data protection laws as explained in the Parents’/Caregivers’ Information Flipbook and Privacy Notice on The Kid's Trial website.**
  - **I understand the data collected during this study will be securely stored for seven (7) years after the completion of the trial.**
  - **I understand that all information I provide will be kept confidential, subject to legal limitations. This means that if my child or I tell the researcher that someone is in immediate danger or risk of harm, the researcher is required to report this to the relevant authorities.**

Yes, I understand and agree to the above statements.

No, I do not agree to the above statements.

- **Consent**

Please answer the following:

**Yes	No**

I am the legal guardian for the child for whom I am giving consent.

I agree that my child can join The Kid's Trial.

**Parent/Guardian Contact Information**

**Parent/Guardian Full Name**

**Participating Child's Full Name**

**Parent/Guardian Email Address**

**Please tell us a bit about your child.**

- **My child is:**

7 years old

8 years old

9 years old

10 years old

11 years old

12 years old

- **What best describes your child's gender?**

A boy A girl

Something not listed here Prefer not to answer

**In which country does your child live?**

**What best describes your child's cultural or ethnic background? This means ‘with what group do they share a background, language, culture or ancestry?**

Please select an option from the drop down menu below. **After selecting an option, another drop down list will appear below.** Select an appropriate option from that list as well. **If you choose to write in a description, you may do so in the next question.**

-- Select --

**You may describe your child's ethnicity here, if you wish.**

- **How likely would you be to let your child take part in a randomised or clinical trial if you were asked?**

Extremely likely Somewhat likely Not sure

Somewhat unlikely Extremely unlikely

#### How did you hear about The Kid's Trial?

Please select an option from the drop down menu below. **After selecting an option, another drop down list will appear below**. Select an appropriate option from that list as well. **If you choose to write-in your referral source, you may do so in the next question.**

-- Select --

**You can tell us more about how you heard about The Kid's Trial here.**

**Please have your child fill out the next sections. You may help if you wish.**

**FOR THE KIDS**

**Both you and your parent or caregiver must agree to take part in The Kid's Trial. If you're both ready, please fill out the form.**

- **Please read the sentences below and check the box to tell us you understand what The Kid’s Trial is and what we are doing. You can** [**find all this information on The Kid's Trial website in the Kids' Information Flipbook.**](https://thekidstrial.ie/)
  - **I have read or listened to the** [**Kid's Information Flipbook**](https://thekidstrial.ie/) **(or had my parent/caregiver explain it to me) on The Kid's Trial website.**
  - **I can stop being part of The Kid's Trial at any time and no one will be upset.**
  - **I understand I will be asked to share my thoughts and opinions when I participate in The Kid's Trial.**
  - **I have talked about joining The Kid's Trial with my parent or caregiver and we have had our questions (if you had any) answered.**
  - **I understand that I (or my parent/caregiver) can contact the research team at any time if we have worries or questions about The Kid's Trial.**

Yes, I understand what the sentences above say, and I agree with them. No, I do not agree with the above sentences.

- **I would like to join The Kid's Trial.**

**Yes****No**

## It’s time for THE KIDS to plan their trial!

Now it’s time to decide how we answer the question the kids chose.

The question is:

“Does sleeping with a comfort item (for example, a soft toy or special blanket) make a difference to how well kids sleep compared to not sleeping with a comfort item?”

- **Should kids in both groups sleep in their usual bed for the entire trial?**

Kids should sleep in their usual bed every night during the trial. If a child has more than one home (for example, boarding school or two

family homes), both places would be their usual beds.

Kids can sleep in places other than their usual bed during the trial, for example, during sleepovers with family or friends, or when traveling.

- **How should kids in both groups tell us how well they slept during the trial?**

**You can choose more than one answer.**

Kids fill out an online survey the day after they finish their trial, telling us how their NIGHTTIME SLEEP was during the trial.

Kids fill out an online survey the day after they finish their trial, telling us how SLEEPY THEY FELT DURING THE DAYTIME during the trial.

Kids fill out an online survey the day after they finish their trial, telling us HOW WELL THEY FELT THEY SLEPT during the trial.

- **Which of these ways of checking sleep do you think is the BEST to show us how well kids slept?**

Kids fill out an online survey the day after they finish their trial, telling us how their NIGHTTIME SLEEP was during the trial.

Kids fill out an online survey the day after they finish their trial, telling us how SLEEPY they felt in the daytime during the trial.

Kids fill out an online survey the day after they finish their trial, telling us HOW WELL THEY FELT THEY SLEPT during the trial.

- **Should kids in the "try-it-out" group use the same comfort item every night, or can they switch items during the trial?**

**Kids in this group can choose any comfort item, such as a teddy bear, a toy, a special piece of clothing, a special blanket, or anything else they find comforting.**

Kids in the "try-it-out" group should use the SAME comfort item each night during their trial.

Kids in the "try-it-out" group can SWITCH which comfort item they use each night during their trial if they wish.

- **When should kids in the "try-it-out" group start using their comfort item each night?**

Kids should start using their comfort item when they begin getting ready for bed each night. For example, if they normally read a book before bed,

they should use their comfort item then and take it with them when they go to bed.

Kids should start using their comfort item when they get into bed.
