# Supplementary material for: Feasibility and Methodological Reflections From the Co-Creation of a Randomized Controlled Trial (The Kid’s Trial): Decentralized, Child-Led Citizen-Science Study
Source: J Med Internet Res. 2026 Aug 28;28:e89240. doi: 10.2196/89240 (PMC13524311; doi:10.2196/89240)
Supplement: Supplementary file 8 [file jmir-v28-e89240-s006.docx]

Running the trial!

(Step 3)

##### CONSENT FORM FOR PARENTS/CAREGIVERS/GUARDIANS

**Research study title: The Kid's Trial: An online randomised trial.**

If you're happy for your child to take part in The Kid's Trial, please fill in this form and help your child fill in their own ASSENT form, too.

Remember, you must fill out a separate consent form for each child in your home who wants to join the trial.

Please use a different email address for each child.

Please make sure you have read or listened to the [Parents' & Caregivers' Information Flipbook](https://thekidstrial.ie/) on the website.

# Please read the following statements:

- **I have reviewed the** [**Parents' & Caregivers' Information Flipbook**](https://thekidstrial.ie/) **and the** [**Privacy Notice**](https://www.thekidstrial.ie/privacynotice) **on the website.**
- **I can withdraw my child from The Kid's Trial at any time without giving any reason.**
- **I have had an opportunity to think about the information in the Flipbook and Privacy Notice and had any questions answered by the Research Team.**
- **I understand my child’s and my own rights in relation to this study.**
- **I understand the data collected during this study will be processed in compliance with data protection laws as explained in the Parents’/Caregivers’ Information Flipbook and Privacy Notice on The Kid's Trial website.**
- **I understand the data collected during this study will be securely stored for seven (7) years after the completion of the trial.**
- **I understand that all information I provide will be kept confidential, subject to legal limitations.**

This means that if my child or I tell the researcher that someone is in immediate danger or risk of harm, the researcher is required to report this to the relevant authorities.

**Yes, I understand and agree to the above statements.**

**No, I do not agree to the above statements.**

# Consent

## Please answer the following:

**Yes	No**

**I am the legal guardian for the child for whom I am giving consent.**

**I agree that my child can join The Kid's Trial.**

**Parent/Guardian Contact Information**

**Parent/Guardian Full Name**

**Participating Child's Full Name**

**Parent/Guardian Email Address**

**Please tell us a bit about your child.**

- **My child is:**

**7 years old**

**8 years old**

**9 years old**

**10 years old**

**11 years old**

**12 years old**

# What best describes your child's gender?

**A boy****A girl**

**An answer not listed here****Prefer not to answer**

# In which country does your child live?

# What best describes your child's cultural or ethnic background? This means ‘with what group do they share a background, language,

**culture or ancestry?**

Please select an option from the drop down menu below. **After selecting an option, another drop down list will appear below.** Select an appropriate option from that list as well. **If you choose to write in a description, you may do so in the next question.**

#### -- Select --

# You may describe your child's ethnicity here, if you wish.

# How likely would you be to let your child take part in a randomised or clinical trial if you were asked?

**Extremely likely****Somewhat likely****Not sure**

**Somewhat unlikely****Extremely unlikely**

### How did you hear about The Kid's Trial?

Please select an option from the drop down menu below. **After selecting an option, another drop down list will appear.** Select an appropriate option from that list as well. **If you choose to write-in your referral source, you may do so in the next question.**

#### -- Select --

# You can tell us more about how you heard about The Kid's Trial here.

**Please have your child fill out the next sections. You may help if you wish.**

**FOR THE KIDS**

**Both you and your parent or caregiver must agree to take part in The Kid's Trial. If you're both ready, please fill out the form.**

- **Please read the sentences below and check the box to tell us you understand what The Kid’s Trial is and what we are doing. You can find all this information on The Kid's Trial website in the** [**Kids' Information Flipbook.**](https://thekidstrial.ie/)
  - **I have read or listened to the** [**Kid's Information Flipbook**](https://thekidstrial.ie/) **(or had my parent/caregiver explain it to me) on The Kid's Trial website.**
  - **I can stop being part of The Kid's Trial at any time and no one will be upset.**
  - **I understand I will be asked to share my thoughts and opinions when I participate in The Kid's Trial.**
  - **I have talked about joining The Kid's Trial with my parent or caregiver and we have had our questions (if you had any) answered.**
  - **I understand that I (or my parent/caregiver) can contact the research team at any time if we have worries or questions about The Kid's Trial.**

**Yes, I understand what the sentences above say, and I agree with them.****No, I do not agree with the above sentences.**

# I would like to join The Kid's Trial.

**Yes****No**

**This is the exciting bit - It’s time for you to join The Kid’s Trial!** Once you know your group, it's important to follow the instructions for all 7 days even if it feels different from your usual routine.

### Remember, no one knows what group you’ll be in, so when you agree to join the trial, be ready to either:

- sleep WITH a comfort item (for example, a soft toy or special blanket, or any other item you choose) for the next 7 nights.

OR

- NOT to sleep with any comfort item for the next 7 nights.

# Before we start, let's see how well you think you usually sleep.

- **Over the past 7 days:**

If you're on a **mobile phone**, move the button to choose from: Never, Almost never, Sometimes, Almost always, Always

#### I was sleepy during the daytime.

#### I had a hard time concentrating because I was sleepy.

#### I had a hard time getting things done because I was sleepy.

#### I had problems during the day because of poor sleep.

## Over the past 7 days, how would you rate your sleep?

This means how well you slept over the past week. Think about how much sleep you got, how easy it was to fall asleep, and if you woke up often at night.

#### Overall sleep

##### Terrible ( 0 ) Poor ( 1, 2, 3 )

##### Fair (this means

**ok) ( 4, 5, 6 )	Good ( 7, 8, 9 )**

##### Excellent ( 10 )

# Do you usually sleep with a comfort item?

Never

Sometimes Always

## This is it! Click the 'NEXT' button to find out which group you're in for The Kid's Trial!

### Wow, congratulations! You got randomised to the 'try-it-out' or 'intervention' group!

This means that, starting tomorrow, for next 7 nights, you should:

- Sleep with a comfort item (you can choose a toy, blanket, piece of clothing, or anything else that you like) every night when you go to bed.
- Use the same comfort item every night.
- Start using your comfort item every night when you are getting ready for bed (for example, if you read before bed, start using your comfort item then).
- Sleep in your usual bed each night.
- Keep everything else about your bedtime the same as usual.
- After 7 days, your grown-up will get an email to help you tell us how you slept.

It's okay if you forget one night, just continue the next night. If you feel very upset or can't sleep, you can stop the trial. Just let your grown-up know.

Remember, by following these instructions carefully, you're helping us learn about sleep and comfort items.

# Got it- see you in 8 days!

### Wow, congratulations! You got randomised to the 'wait-and-see' or 'control' group!

This means that, starting tomorrow, for the next 7 nights, you should:

- NOT sleep with any comfort items when you go to bed each night.
- Sleep in your usual bed each night.
- Keep everything else about your bedtime the same as usual.
- After 7 days, your grown-up will get an email to help you tell us how you slept.

It's okay if you forget one night, just continue the next night. If you feel very upset or can't sleep, you can stop the trial. Just let your grown-up know.

Remember, by following these instructions carefully, you're helping us learn about sleep and comfort items.

**Got it- see you in 8 days!**
