# Supplementary material for: Feasibility and Methodological Reflections From the Co-Creation of a Randomized Controlled Trial (The Kid’s Trial): Decentralized, Child-Led Citizen-Science Study
Source: J Med Internet Res. 2026 Aug 28;28:e89240. doi: 10.2196/89240 (PMC13524311; doi:10.2196/89240)
Supplement: Supplementary file 9 [file jmir-v28-e89240-s007.docx]

# Sharing the results!

## Step 5

CONSENT FORM FOR PARENTS/CAREGIVERS/GUARDIANS

Research study title: The Kids Trial: An online randomised trial.

If you're happy for your child to take part in The Kid's Trial, please fill in this form and help your child fill in their own ASSENT form, too.

Remember, you must fill out a separate consent form for each child in your home who wants to join the trial. Please use a different email address for each child.

Please [make sure you have read or listened to the Parents' & Caregivers' Information Flipbook on the website.](https://thekidstrial.ie/)

- **Please read the following statements:**
  - **I have reviewed the** [**Parents' & Caregivers' Information Flipbook**](https://thekidstrial.ie/) **and the** [**Privacy Notice**](https://www.thekidstrial.ie/privacynotice) **on the website.**
  - **I can withdraw my child from The Kid's Trial at any time without giving any reason.**
  - **I have had an opportunity to think about the information in the Flipbook and Privacy Notice and had any questions answered by the Research Team.**
  - **I understand my child’s and my own rights in relation to this study.**
  - **I understand the data collected during this study will be processed in compliance with data protection laws as explained in the Parents’/Caregivers’ Information Flipbook and Privacy Notice on The Kid's Trial website.**
  - **I understand the data collected during this study will be securely stored for seven (7) years after the completion of the trial.**
  - **I understand that all information I provide will be kept confidential, subject to legal limitations. This means that if my child or I tell the researcher that someone is in immediate danger or risk of harm, the researcher is required to report this to the relevant authorities.**

**Yes, I understand and agree to the above statements.****No, I do not agree to the above statements.**

- **Consent**

Please answer the following:

**Yes	No**

**I am the legal guardian for the child for whom I am giving consent.**

**I agree that my child can join The Kid's Trial.**

Parent/Guardian Contact Information

**Parent/Guardian Full Name**

**Participating Child's Full Name**

**Parent/Guardian Email Address**

- **My child is:**

**7 years old**

**8 years old**

**9 years old**

**10 years old**

**11 years old**

**12 years old**

- **What best describes your child's gender?**

**A boy****A girl**

**Something not listed here****Prefer not to answer**

Please have your child fill out the next sections. You may help if you wish.

FOR THE KIDS

Both you and your parent or caregiver must agree to take part in The Kid's Trial. If you're both ready, please fill out the form.

- **Please read the sentences below and check the box to tell us you**

understand what The Kid’s Trial is and what we are doing. You can [find all this information on The Kid's Trial website in the Kids' Information Flipbook.](https://thekidstrial.ie/)

- **I have read or listened to the** [**Kid's Information Flipbook**](https://thekidstrial.ie/) **(or had my parent/caregiver explain it to me) on The Kid's Trial website.**
- **I can stop being part of The Kid's Trial at any time and no one will be upset.**
- **I understand I will be asked to share my thoughts and opinions when I participate in The Kid's Trial.**
- **I have talked about joining The Kid's Trial with my parent or caregiver and we have had our questions (if you had any) answered.**
- **I understand that I (or my parent/caregiver) can contact the research team at any time if we have worries or questions about The Kid's Trial.**

**Yes, I understand what the sentences above say, and I agree with them.****No, I do not agree with the above sentences.**

- **I would like to join The Kid's Trial.**

**Yes****No**

## It's time for you to tell us how we should share the results of The Kid's Trial!

It’s really important that the results of randomised and clinical trials are shared. How else can anyone learn what has been discovered? Even if the results were not what the researchers thought they would be, it’s important that we share what we found!

Please tell us how you think we should share the results with the world.

Below, we have listed a few different ways we can share the results. Please put these in order from your most favourite (at the top) to your least favourite (at the bottom).

Drag your choices here to rank them

Least favourite

Most favourite

**Use a short report in words that kids can understand.**

**Use a scientific report that helps researchers understand The Kid's Trial.**

**Use a video report given by the project leader of The Kid’s Trial.**

**Use an animated video.**

**Use a longer, detailed report in words that kids can understand.**

**Using pictures, like charts and graphs.**

- **Below, we have listed a few places we could share the results. Please put these in order from your most favourite (at the top) to your least favourite (at the bottom).**

Drag your choices here to rank them

Least favourite

Most favourite

**A podcast.**

**Other media, like radio.**

**Print media, like newspapers.**

**The Kid’s Trial YouTube channel.**

**The Kid’s Trial website.**

**Social networking sites, like TikTok, Instagram, X, and Facebook.**

We are really excited to hear what you might have learned about trials.

Please tell us how much you agree with these sentences.

- **I understand what a randomised trial is.**

Disagree	Do not agree or disagree

Agree

I understand why randomised trials or clinical trials are important.

Disagree	Do not agree or disagree

Agree

- **I understand how researchers do randomised trials.**

Disagree	Do not agree or disagree

Agreed

We want to hear what you thought of The Kid’s Trial, so tell us how you felt about the project!

Please tell us how much you agree with these sentences.

- **Overall, taking part in The Kid’s Trial was fun.**

Disagree	Do not agree or disagree

Agree

- **The videos on the website helped me understand the project.**

Disagree	Do not agree or disagree

Agree

- **The written information on the website helped me understand the project.**

Disagree	Do not agree or disagree

Agree

- **Taking part in The Kid's Trial made me more interested in health research.**

Disagree	Do not agree or disagree

Agree

Do you have anything else you would like to say about taking part in The Kid’s Trial? If you do, you can tell us here.
